# Inhibiting autophagy enhances anti-cancer properties of sulforaphane

**DOI:** 10.1038/s41598-026-35891-x

**Published:** 2026-01-15

**Authors:** Marta Zarzycka, Małgorzata Kotula-Balak, Dorota Gil

**Affiliations:** 1 Chair of Medical Biochemistry, Jagiellonian University Medical College, Kopernika 7, 31-034 Kraków, Poland; 2 Department of Basic Sciences, Faculty of Veterinary Medicine, University of Agriculture in Krakow, Mickiewicza 24/28, 30-059 Krakow, Poland

**Keywords:** ICAM-1, Bladder cancer, Autophagy, Sulforaphane, Chloroquine, Cancer, Oncology

## Abstract

Bladder cancer (BC) has an extremely low survival rate due to its tendency to metastasize. The antimalarial drug chloroquine (CQ) can inhibit BC progression and invasiveness by targeting basal autophagy. However, the mechanism by which CQ affects BC is not defined. Here, we revealed that although CQ showed an anticancer effect by reducing the migration and proliferation of the analyzed bladder cancer cells, it increased the expression of ICAM-1, a protein whose expression is associated with higher tumorigenic potential. Sulforaphane (SFN), a well-known ICAM-1 inhibitor, significantly contributed to the enhancement of anticancer effect of CQ, through regulation of both AKT/GSK-3β and mTOR/ULK pathways, which led to effective inhibition of autophagy, reduced proliferation level or inhibition of migration of the analyzed bladder cells. Moreover, regulation of pathways related to autophagy contributed to the reduction of mitochondrial membrane potential and regulation of ROS level. Nevertheless, the level of influence of CQ and SFN on the anticancer effects strongly depended on the molecular basis of the analyzed bladder cell lines. Our data indicate that although CQ exerts antitumor effects on bladder cancer cells, it should be noted that activation of some pro-tumor pathways may be associated with subsequent disease relapse or treatment resistance.

**Supplementary Information:**

The online version contains supplementary material available at 10.1038/s41598-026-35891-x.

## Introduction

Bladder cancer (BC) is currently one of the most common malignant tumors of the genitourinary system, with additional gender differences in incidence and prognosis^1^, classified into “molecular subtypes” with multiple pathogenic pathways depending on whether the disease is non-muscle invasive or muscle invasive^2^. Specific genomic alterations enriched in particular molecular subtypes may include mutations targeting genes involved in numerous cellular processes^1^, which may lead to the disruption of target proteins’ functioning and thus to deregulation of signaling pathways. It is consistent with the studies conducted on bladder cancer cell lines, which revealed different sensitivities to androgens, depending on the set of activated genes involved in metastasis^3^. Similarly, studies using the same bladder cancer cell lines revealed that silencing of ICAM1 induced different anticancer responses, which correlated with *N*-cadherin’s expression level^4^. The results of the above studies indicate that the antitumor response in bladder cancer is closely dependent on the set of activated genes, which may be involved in cancer progression. Autophagy is a conserved cellular process that plays an important role in adaptation to starvation, development, and cell death, thus, the involvement of its defects in different human diseases has been widely described^5,6^. In cancer biology, autophagy plays a complex and stage-specific role and promotes multiple steps during cancer progression. Indeed, during the initial stage of cancer development, the influence of autophagy on tumor suppression by maintaining genomic integrity and preventing inflammation and proliferation was revealed^7^. However, after the establishment of cancer, aberrant autophagy may enable the spread of cancer progenitor cells, aiding in the proliferation of oncogenic cells and continued tumor progression. Hence, understanding the role of autophagy in cancer progression and drug resistance is important for designing novel strategies to improve current therapies^8^. Chloroquine (CQ) is a well-known anti-cancer agent that successfully block autophagy functions to inhibit cancer invasiveness^9^. The anti-cancer effect of chloroquine has been observed alone or in combination with other anticancer agents in various types of cancer^10–12^. Inhibition of lysosome activity by chloroquine arrests the latter step of autophagy, degradation of the autolysosome, which fails to provide energy through the autophagy pathway. Moreover, it seems that the pharmacological anticancer activity of chloroquine also involves other mechanisms, such as inhibition of proliferation or induction of apoptosis^9–12^. The multi-faceted anti-cancer action of CQ makes this drug attractive for cancer therapy. Moreover, the treatment combination with autophagy inducers and inhibitors in cancer cells enhances the effect of anti-cancer therapies^13,14^. For this reason, we decided to evaluate CQ influence on bladder cancer also in combination with sulforaphane. Sulforaphane (SFN) is a naturally occurring isothiocyanate derived from cruciferous vegetables such as broccoli, which has been reported to exhibits a broad spectrum of anticancer effects. It inhibits the growth of a variety of cancers, such as breast, prostate, colon, skin, lung, gastric, or bladder cancer^15^, induce apoptosis, inhibits survival pathways, influences autophagy and modulates oxidative stress responses^16^. Based on the results so far, the effect of SFN is not completely clear, as it was able to both activate and inhibit autophagy^17^. However, researches showed that SFN and its analogs inhibit the fusion of autophagosome to lysosome and caused the accumulated autophagy flux LC3 II/I leading to apoptosis^18,19^. On the other hand, SFN can influence the mTOR pathway, disrupting autophagy regulation and suppressing this mechanism^20^. The other possible anticarcinogenic activity of SFN can be related to its ability to decrease of ICAM-1 expression level. According to our recent studies^4^, ICAM-1 exhibited the oncogenic role in bladder cancer cells; thus in the present study is a continuation of the previous ones. We would like to expand our knowledge about the molecular signaling of regulated by ICAM-1 in bladder cancer in combination with autophagy regulation, which will be helpful to identify the potential molecular targets for bladder cancer treatment. Since SFN has the ability to enhance the cytotoxicity of chemotherapy on cancer cells while potentially minimizing damage to normal cells and healthy tissues^21^, we decided to test its effect in combination with CQ on bladder cancer.

## Materials and methods

### Cell culture and treatment

T24 (transitional cancer cells of the urine bladder), HTB9 (urinary bladder cancer grade II), and HT1376 (urinary bladder carcinoma, grade III) cell lines were obtained from the American Type Culture Collection. The cell lines were purchased from ATCC and authenticated by short tandem repeat analysis by LGC Standards. Moreover, all cell lines were examined for mycoplasma infection by PCR analysis. Cells were cultured in RPMI-1640 medium supplemented with 10% fetal calf serum (FCS, Gibco) and 1% penicillin/streptomycin^22^ at 37 °C in a humidified atmosphere of 5% CO_2_. At 75–80% confluency, cells were treated with sulforaphane (SFN)-10µM (Sigma-Aldrich), chloroquine (CQ) − 50µM alone and in combination. DMSO was added to the reference cells. The concentration of CQ was selected based on our previous experience^12,23^, but of SFN based on the proliferation levels (Fig. 2 in supp materials) and literature data^24^. After 24 h treatments, cultured cells were washed twice with ice-cold PBS, and the cells were lysed by adding ice-cold lysis buffer (0.0625 M Tris/HCl pH 6.8, 2%SDS, 10% glycerol, 5%β-mercaptoethanol), as described previously^4^.

### Western blot analysis

Cell lysates containing equal amounts of protein were separated on 10%SDS-PAGE gels and subsequently transferred onto a PVDF membrane^4^. All primary antibodies used in western blot analysis are described in Table 1. The presence of the primary antibody was revealed with horseradish peroxidase-conjugated secondary antibodies diluted 1:3000 (Cell Signalling Technology Inc) and visualized with an Enhanced Chemiluminescence (ELC)^25^. All immunoblots were stripped with stripping buffer containing 2 mM glycine–HCl, pH 2, 1% (wt/v) SDS for 30 min and incubated in antibody against β-actin (dilution, 1:3000; Sigma-Aldrich, St.Louis, MO), which served as a loading control. To obtain quantitative results, immunoblots were scanned using the public domain ImageJ software (NIH).

**Table 1:** Details of primary antibodies used for Western blot analysis.

Primary antibody		Host species	Dilution (application)	Vendor
ICAM-1		Rabbit	1:1000	Cell signaling technology
N-cadherin		Rabbit	1:1000	R&D system
β-catenin		Mouse	1:1000	BD transduction laboratories
β-catenin S552		Rabbit	1:1000	Cell signaling technology
β-catenin T41, S33/37		Rabbit	1:1000	Cell signaling technology
AKT		Mouse	1:1000	BD transduction laboratories
AKT S473		Rabbit	1:1000	Cell signaling technology
GSK-3β		Mouse	1:1000	BD transduction laboratories
GSK-3β Y 216		Mouse	1:1000	BD transduction laboratories
GSK-3β S9		Rabbit	1:1000	Cell signaling technology
p62/SQSTM1		Rabbit	1:1000	Cell signaling technology
LC3A/B		Rabbit	1:1000	Cell signaling technology
ULK		Rabbit	1:1000	Cell signaling technology
ULK S757		Rabbit	1:1000	Cell signaling technology
ULK S555		Rabbit	1:1000	Cell signaling technology
mTOR		Rabbit	1:1000	Cell signaling technology
mTOR S2448		Rabbit	1:1000	Cell signaling technology
Cyclin D_3_		Mouse	1:1000	Cell signaling technology
β-actin		Mouse	1:12,000	Sigma-Aldrich

### Proliferation

The proliferation of cells was assessed with the crystal violet test as previously described^26^. The absorbance at 450 nm of each sample was measured by a microplate reader (BioKom Synergy, HTX). Each group had 3 replicates.

### In vitro wound healing

The in vitro wound healing model was used to compare the migration of bladder cell lines after SFN, CQ and the combination of CQ with SFN treatment. Cells were grown until confluent in 24-well plates for the wound healing assay. A small linear scratch was created in the confluent monolayer by gently scraping it with a sterile 1 mL pipette tip. Twenty-four hours later, images of the migrated cells were taken using a digital camera (Nikon, Tokyo, Japan), connected to the inverted microscope. The assay will be repeated thrice in duplicate.

### Mitochondrial membrane potential and reactive oxygen species production

Cells were plated at 2 × 10^4^cells/well in triplicate, in 24-well plates, and treated with CQ or SFN or a combination of SFN and CQ the next day for 24 h. Rhodamine123 (membrane-potential-dependent dye) was applied to monitor mitochondrial function. Rhodamine was used as previously described^27^.

Dihydrorhodamine123 (DHR123) is an uncharged and nonfluorescent indicator of reactive oxygen species (ROS), which is oxidized to cationic rhodamine123 in the presence of reactive oxygen species. DHR123 was prepared with a 1mM stock solution in DMSO. After staining and rinsing the cells three times in PBS, fresh prewarmed media were added and measured on a plate reader (BioKom). The quantitative analysis measured the fluorescence of cells at excitation 485/20 nm/emission 528/20 nm, sensitivity75 ^12,27^.

### Statistical analysis

The Shapiro–Wilk W-test was used to check the normality of each variable. Levene’s test was used to assess the homogeneity of variance. Statistical analyses of data from in vitro studies were performed by one-way analysis of variance (ANOVA), followed by Dunnett’s post hoc comparison test to determine which values differed significantly from the controls. All analyses were made using Statistica13 software (StatSoft Inc., Tulsa, OK, USA). All experiments were repeated at least three times, and each experiment was performed in triplicate (*n* = 9) to mean and standard deviation (± SD) values. Data were considered statistically significant at *p < 0.05, **p < 0.01, ***p < 0.001.

## Results

### ICAM-1 expression and the signaling pathway regulation

**Fig. 1:**
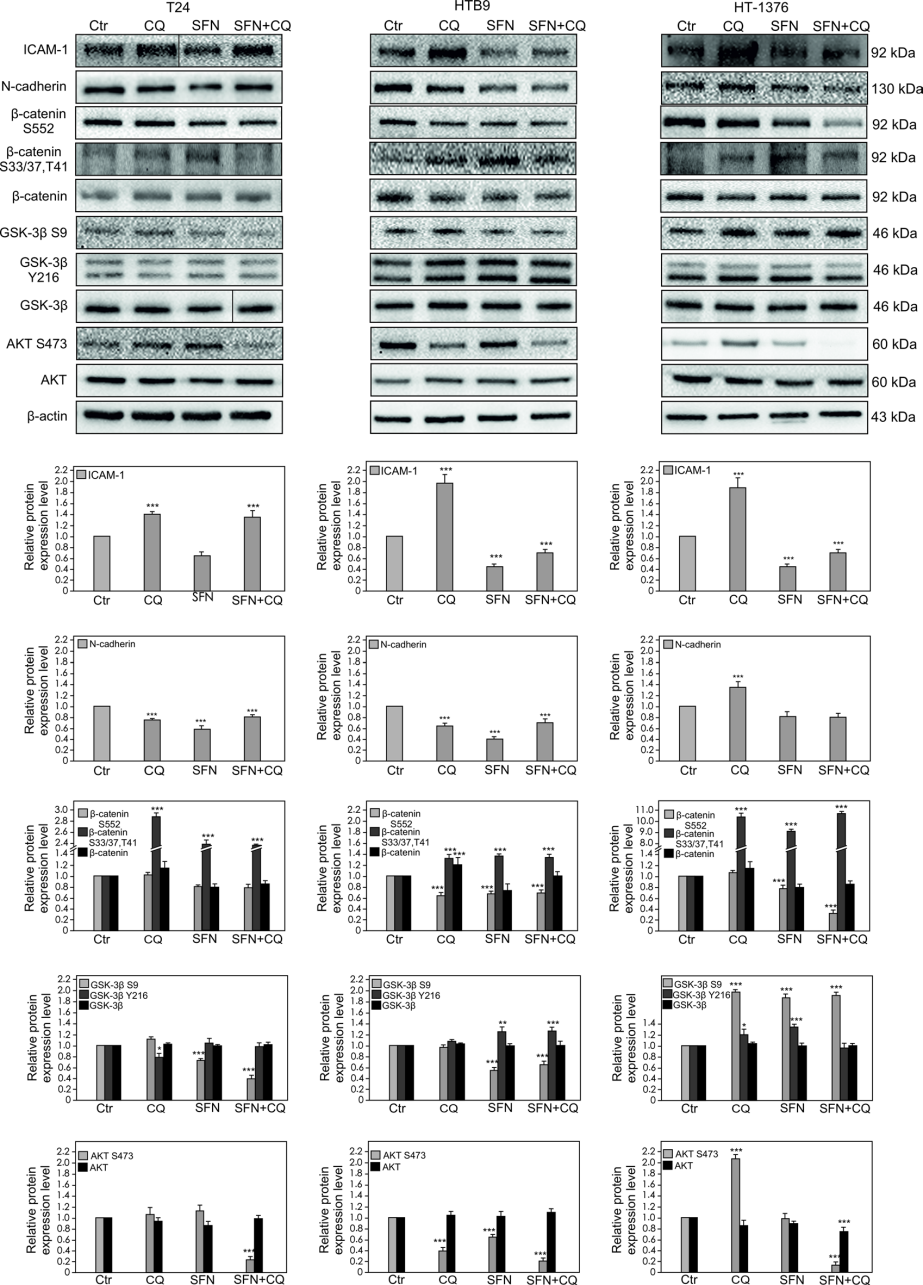
Effect of Chloroquine (CQ), Sulforaphane (SFN) or Chloroquine in combination with Sulforaphane on the ICAM-1, N-cadherin, β-catenin, GSK-3β, AKT expression and β-catenin S552, β-catenin S33/37, T41, GSK-3β S9, GSK-3β Y216, AKT S473 phosphorylation levels. The expression and phosphorylation level were determined by Western blotting. Representative blots are displayed. Vertical black line indicates the place where the blots from separate gels were connected. The histograms quantitatively represent data after densitometry (mean ± SD) of three independent experiments. Each data point was normalized against its corresponding β-actin data point. β-actin was used as a protein loading control. Asterisks indicate significant differences from control cells. Values are denoted as *p < 0.05, *p < 0.01**, *p* < 0.001***.

In all analyzed cancer cell lines, the incubation with CQ contributed to the significant increase of ICAM-1 expression. However, CQ influence on N-cadherin expression strongly dependent on the analyzed bladder cell line. In T24 the expression of N-cadherin is slightly decreased, in HTB-9 significantly decreased, and in HT-1376 significantly increased. Treatment with SFN decreased the expression of both ICAM-1 and N-cadherin, but combination of CQ with SFN maintained the reduced expression level of both, ICAM-1 and N-cadherin only in HTB9 and HT-1376 cell lines (Fig. 1). Treatment with CQ contributed to decreasing of β-catenin phosphorylation level on S552 residue only in HTB9 cell line. However, in the case of treatment with SFN, and in combination of CQ with SFN the decreased phosphorylation level of β-catenin on S552 was detected in all analyzed cell lines. Interestingly, SFN and CQ significantly increased phosphorylation of β-catenin on S33/37, which is associated with the β-catenin degradation in the proteosome in all analyzed bladder cancer cell lines (Fig. 1).

Our study revealed that in all analyzed bladder cell lines the activity of GSK-3β was down-regulated after treatment with CQ, what was evaluated based on the increased phosphorylation of GSK-3β on S9. Treatment with SFN and its combination with CQ also contributed to down-regulation of GSK-3β but only in HT-1376 cell line. Sulforaphane and its combination with CQ up-regulated GSK-3β activity in T24 and HTB9. Moreover, GSK-3β activity is up-regulated by autophosphorylation at Y216, what was observed after treatment with SFN in T24 cell line, and in all analyzed treatment combinations in HTB-9 cell (Fig. 1). The principal regulator of GSK-3β, AKT kinase was up-regulated by its phosphorylation of S473 residue, what was observed after treatment with CQ in T24 and HT-137 cell lines. In T24 the increased level of AKT phosphorylation was not statistically significant. Moreover, decreased phosphorylation level was demonstrated after treatment with CQ and SFN alone in HTB-9 cell line, and after incubation with a combination of CQ with SFN in all analyzed bladder cancer cells (Fig. 1).

### mTOR/ULK signaling pathway regulation

**Fig. 2:**
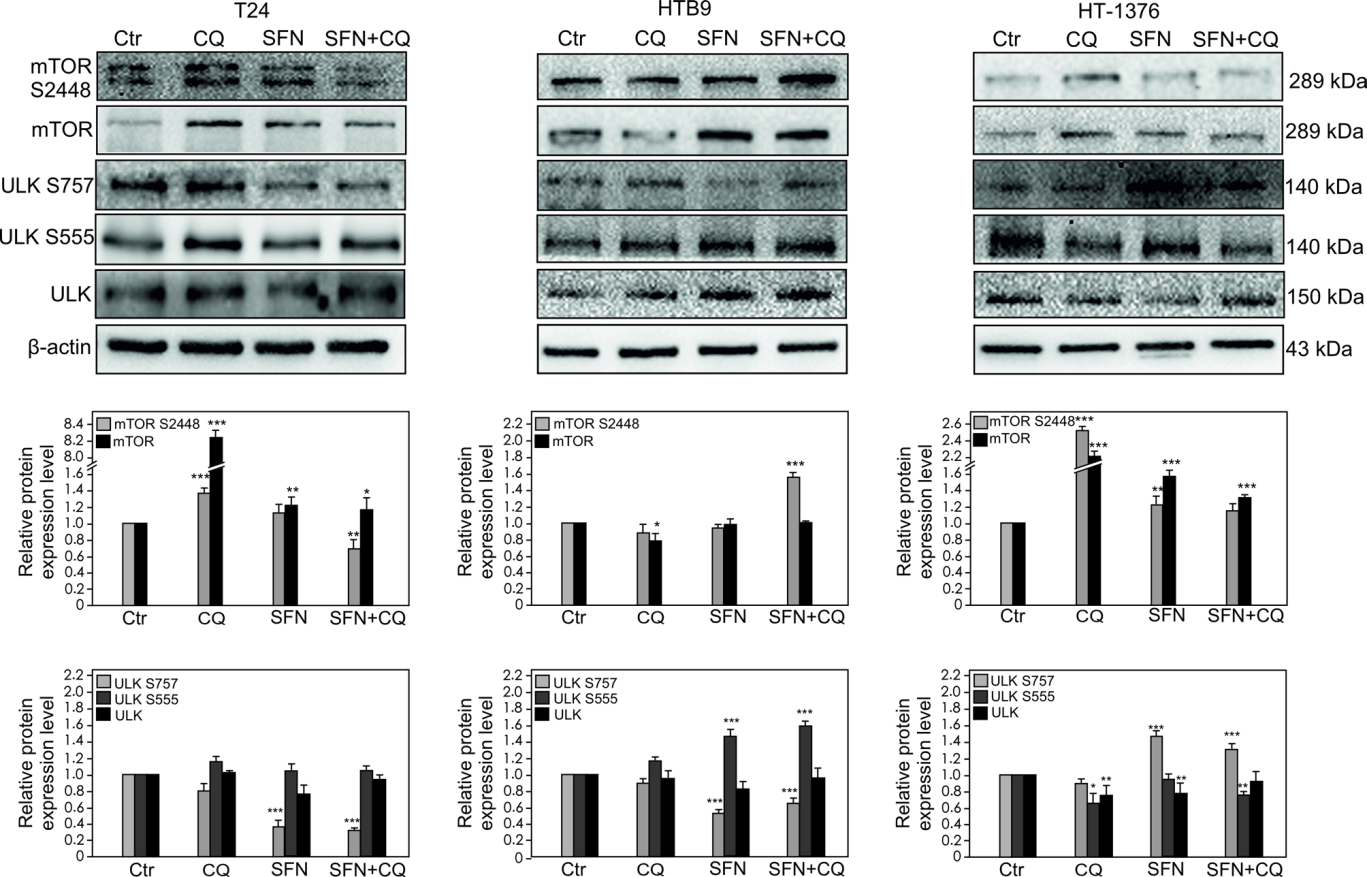
Effect of Chloroquine (CQ), Sulforaphane (SFN) or Chloroquine in combination with Sulforaphane on the mTOR and ULK expression and mTOR S2448, ULK S757 and ULK S555 phosphorylation levels. The expression and phosphorylation level were determined by Western blotting. Representative blots are displayed. The histograms quantitatively represent data after densitometry (mean ± SD) of three independent experiments. Each data point was normalized against its corresponding β-actin data point. β-actin was used as a protein loading control. Asterisks indicate significant differences from control cells. Values are denoted as *p < 0.05, *p < 0.01**, *p* < 0.001***.

mTOR can inhibit autophagy and its phosphorylation on S2448 is implicated in mTOR activity and suppression^28^, thus expression and phosphorylation level of mTOR were analyzed (Fig. 2). We found that in T24 and HT-1376 cell lines mTOR S2448 level increased significantly after treatment with CQ. In the case of HTB9 significant mTOR phosphorylation level was only observed after treatment with CQ and SFN combination. Active mTORC1 may phosphorylate ULK on S757 to prevent ULK interaction with and activation by AMPK. Our data revealed that phosphorylation of ULK on S757 was correlated with the level of mTOR on S2448 in HT1376. In the case of ULK on S555, the elevated level was demonstrated after treatment with CQ, SFN and in combination of CQ and SFN only in HTB9 cancer cell lines. In HT-1376 bladder cancer cells, CQ contributed to the decreased level of ULK S555.

### Autophagy regulation

**Fig. 3:**
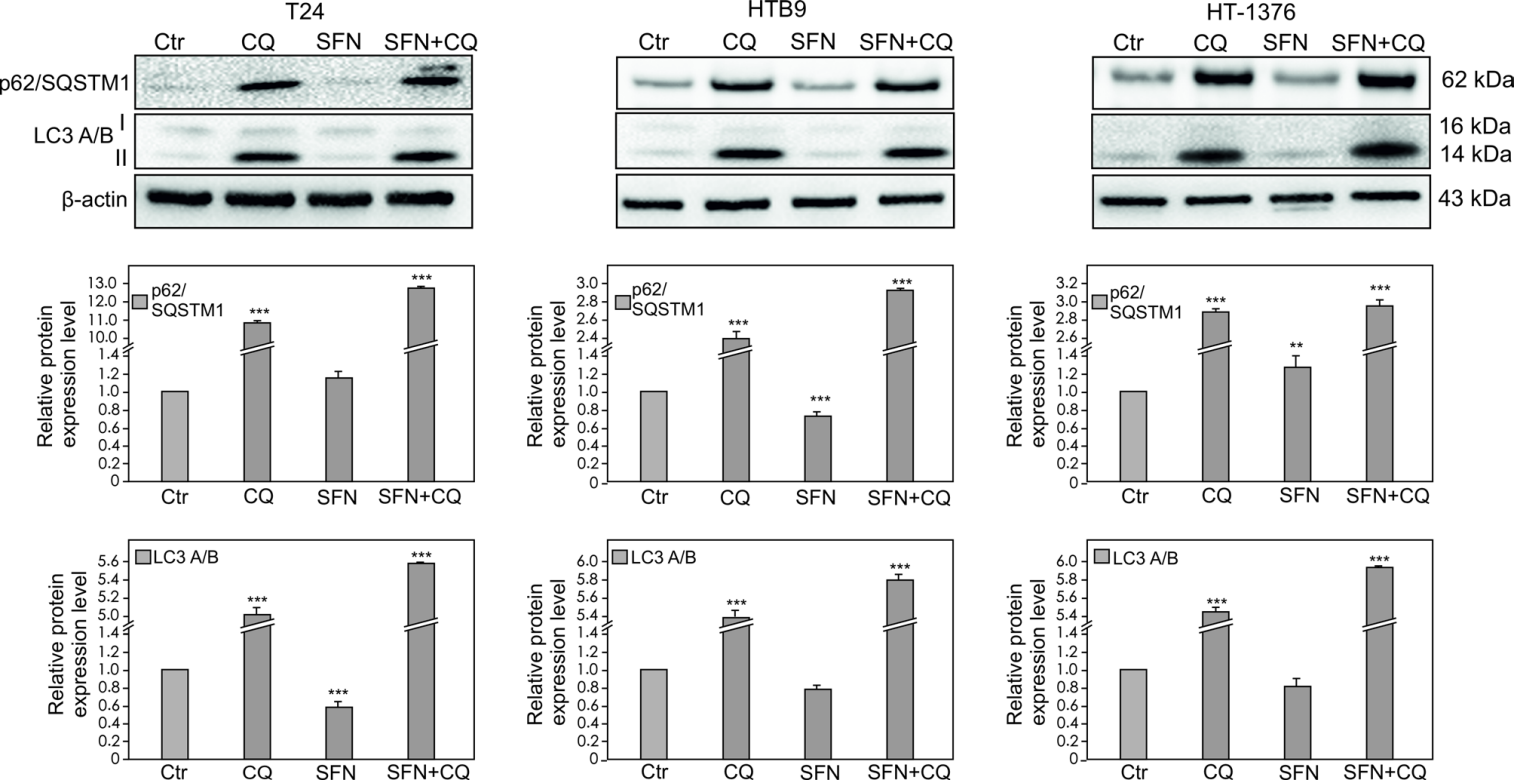
Effect of Chloroquine (CQ), Sulforaphane (SFN) or Chloroquine in combination with Sulforaphane on the p62/SQSTM1 and LC3 A/B expression level. The expression and phosphorylation level were determined by Western blotting. Representative blots are displayed. The histograms quantitatively represent data after densitometry (mean ± SD) of three independent experiments. Each data point was normalized against its corresponding β-actin data point. β-actin was used as a protein loading control. Asterisks indicate significant differences from control cells. Values are denoted as *p < 0.05, *p < 0.01**, *p* < 0.001***.

In all analyzed bladder cancer cell lines, high basal autophagy activity was evaluated by detecting two canonical autophagy markers: LC3-II and p62/SQSTM1^29^. As a treatment with CQ inhibits lysosomal activity and arrests the latter step of autophagy, an increased level of LC3-II as well as p62/SQSTM1 is observed. Thus, our data revealed that CQ inhibited autophagy in all analyzed cancer cell lines (Fig. 3).

### Mitochondrial functioning

**Fig. 4:**
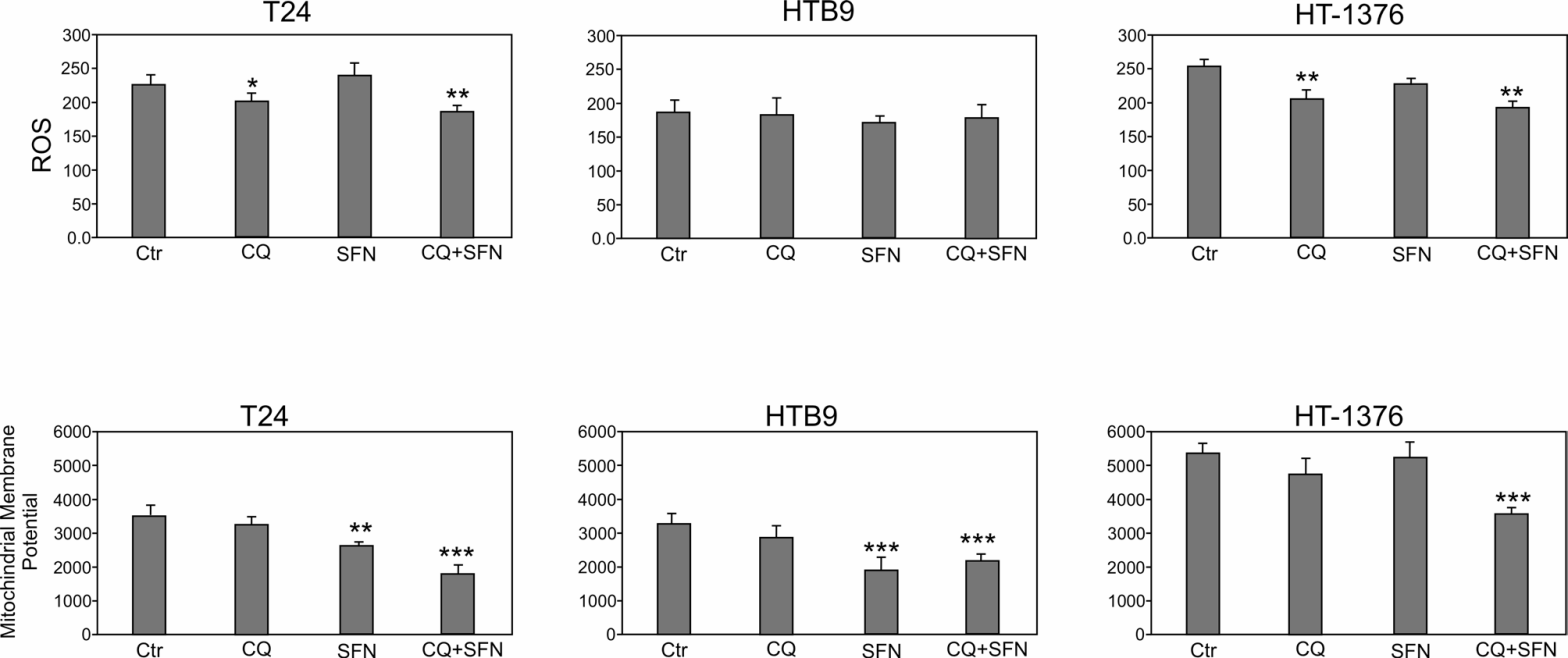
Effect of Chloroquine (CQ), Sulforaphane (SFN) or Chloroquine in combination with Sulforaphane on mitochondrial condition: Dihydrorhodamine 123 (DHR 123) is a reactive oxygen species (ROS) indicator and Mitochondrial membrane potential quantification using Rhodamine 123. The quantitative analysis measured the ‘sample’s fluorescence at excitation 485/20 nm/emission 528/20 nm, sensitivity 75. The histograms are a quantitative representation of data (mean ± SD) from three independent experiments. Asterisks indicate significant differences from control cells. Values are denoted as *p < 0.05, **p < 0.01, and ***p < 0.001.

Mitochondria are key organelles for energy production and regulate cellular redox signaling pathways and programmed cell death^30^. To evaluate the effects of CQ and SFN on mitochondrial function, we checked the production of the mitochondrial membrane potential and reactive oxygen species (ROS) (Fig. 4). In our study, CQ decreased the normal mitochondrial functionality and suppressed the generation of ROS in all analyzed bladder cancer cell lines. However, slight decrease in ROS level was demonstrated in HTB9 cells. Sulforaphane contributed to significant decrease of mitochondrial membrane potential only in T24 and HTB9. In the case of ROS generation, differences in the level were observed across analyzed bladder cancer cells. Increased level was observed in T24, decreased level was demonstrated in HT-1376, however no difference was detected in HTB-9 bladder cancer cells line (Fig. 4).

### Proliferation

**Fig. 5:**
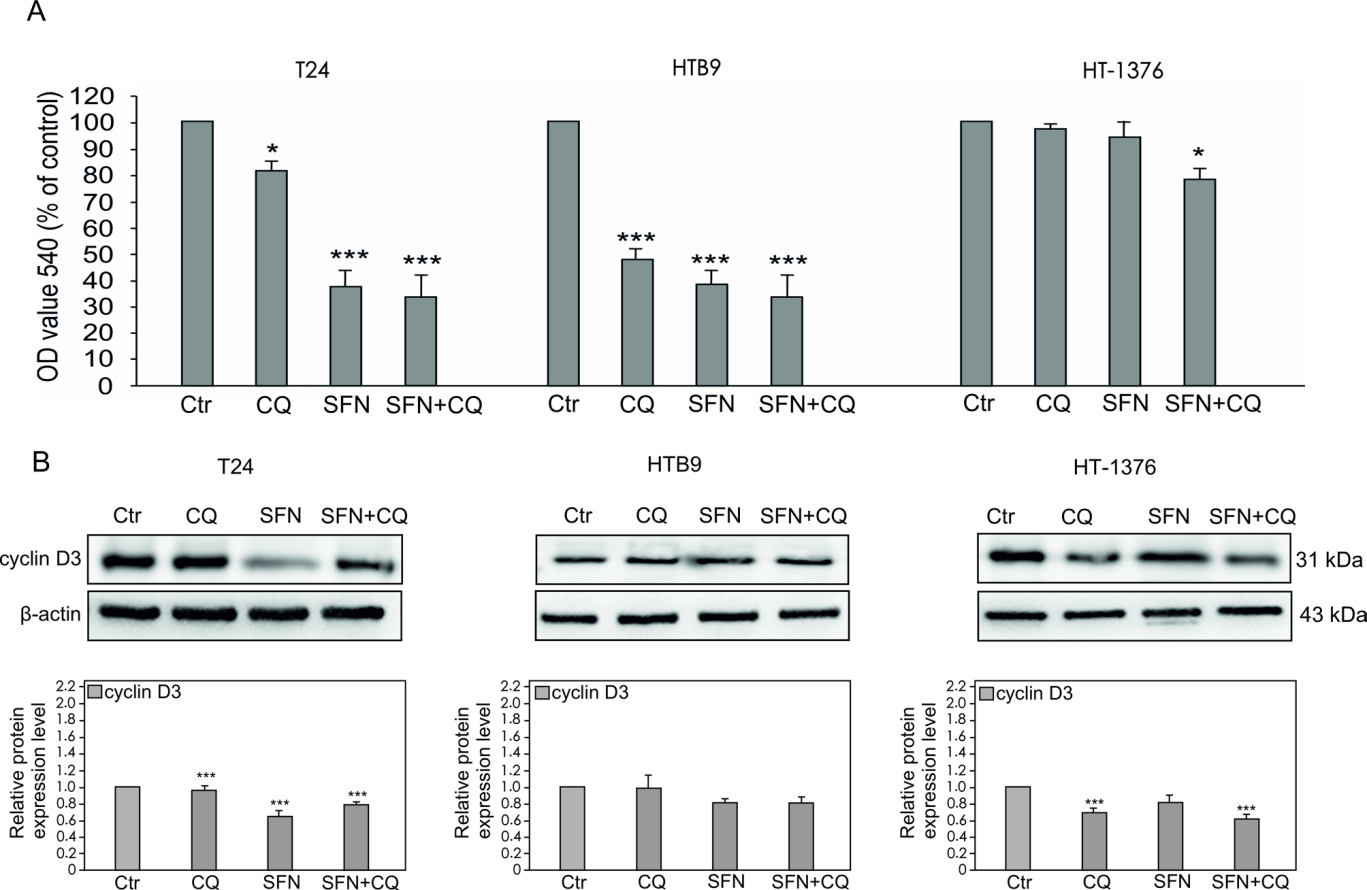
Effect of Chloroquine (CQ), Sulforaphane (SFN) or Chloroquine in combination with Sulforaphane on the survival of bladder cancer cells. Values are expressed as mean ± standard deviation in 6 wells in three independent experiments; an asterisk (*) indicates a significant difference: *p < 0.05, **p < 0.01, ***p < 0.001. (**A**) Cell proliferation was assessed with the crystal violet test. All results are presented as % of control (Ctr). (**B**) The expression of cyclins detected by Western blotting. Representative blots and quantitative representation of data after densitometry (mean ± SD) of three independent experiments on histograms are displayed. β-actin was used as a protein loading control.

The most visible effect of SFN or/and CQ treatment on proliferation level was observed in HTB9 (Fig. 5A). However, SFN alone and SFN in combination with CQ contributed to a significant decrease in the proliferation level in T24 cancer cell lines. In the case of HT-1376 statistically decreased level of proliferation was observed only after treatment with a combination of SFN with CQ (Fig. 5A). In analyzed bladder cancer cell lines differences in responses after CQ or SFN treatment are visible in the expression of cyclin D3, for which a statistically lower expression level was observed in the T24 cell line after SFN treatment and in the HT-1376 line after CQ treatment (Fig. 5B).

### Cell migration

**Fig. 6:**
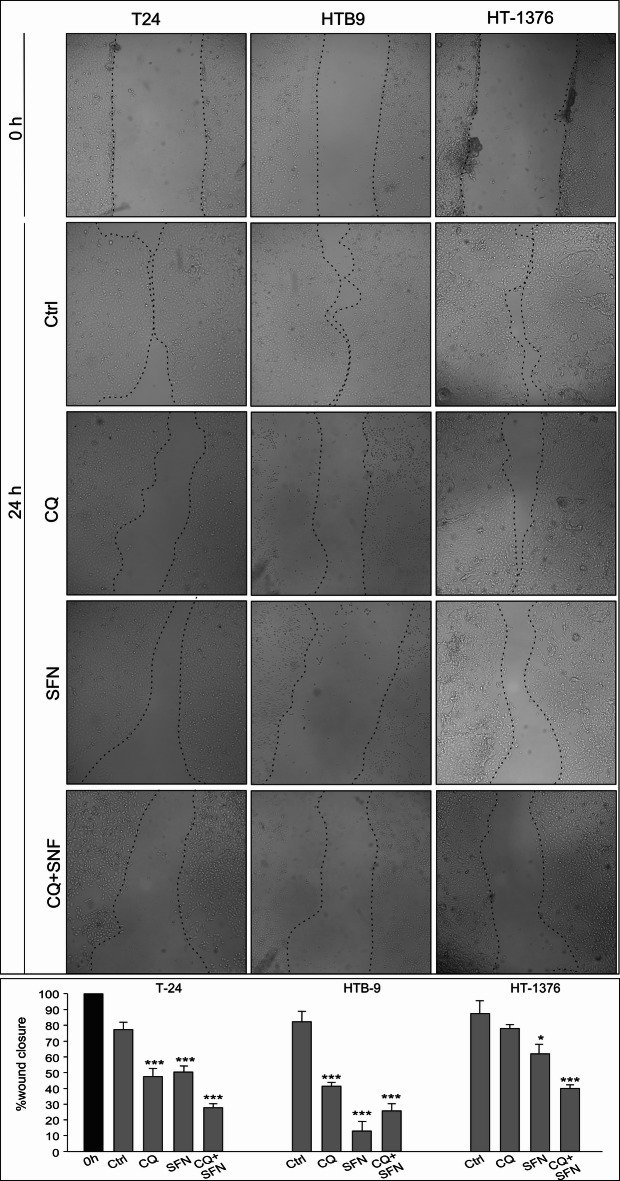
Effect of Chloroquine (CQ), Sulforaphane (SFN) or Chloroquine in combination with Sulforaphane on the bladder cancer migration. In vitro wound healing/migration assay, the confluent cell monolayer was wounded, and after 24 h, the wound closure was captured by a digital camera connected to the inverted microscope. The assay was repeated thrice. The wound closure area was measured using ImageJ and expressed as the percentage of wound closure. The histograms are a quantitative representation of data (mean ± SD) from three independent experiments. Asterisks indicate significant differences from control cells. Values are denoted as *p < 0.05, **p < 0.01, and ***p < 0.001.

To evaluate the effects of SFN, CQ, and SFN in combination with CQ on bladder cancer cell migration, we performed a scratch wound healing assay (Fig. 6). The results show that significantly reduced wound closure in T24 and HTB9 bladder cancer cell lines was observed in all analyzed cases. However, in HTB9 the most effective effect was detected after treatment with SFN, but in T24, after treatment with a combination of SFN and CQ. In the case of HT-1376 significant effect on cell migration was observed only after treatment SFN in combination with CQ.

## Discussion

Intercellular adhesion molecule 1 (ICAM-1) is a 90 kDa glycosylated transmembrane protein of the immunoglobulin superfamily. ICAM-1 plays an important role in the adhesion of immune cells to endothelial cells^31^. Numerous studies have indicated a correlation between elevated ICAM-1 expression and inflammatory disorders, including multiple sclerosis^32–34^. However, we identified the cell surface protein ICAM-1 as a potential candidate for targeted metastatic bladder cancer therapy^4^. Our research uncovered ICAM-1’s critical role in promoting the metastatic potential. Specifically, we found that bladder cancer cells exhibit elevated ICAM-1 expression in correlation with high levels of N-cadherin, thus, we supposed its role in inducing epithelial-mesenchymal transition (EMT). These findings highlight ICAM-1’s potential as both a prognostic marker and a therapeutic target in bladder cancer.

To extend our knowledge regarding ICAM-1 to fundamental processes related to carcinogenesis, in the present study, we aimed to elucidate its role in the autophagy-dependent regulation of cell signaling. As it known, molecular pathways linking cancer progression and autophagy modulation are tightly connected^35,36^. This is especially relevant in the human bladder cancer, which exhibits a high basal level of autophagic activity^29^. It is noteworthy that the dependence of tumor cells on autophagy varies in different tumors. Several studies have suggested that the effect of autophagy on the destiny of tumor cells depends on the type of tumor, genetic context, tumor stage^37^ and tumor microenvironment^38^.

Chloroquine, an anti-malarial drug, is an autophagic inhibitor which blocks autophagosome fusion with lysosome and slows down lysosomal acidification^39^. Recently, the ability of CQ to block autophagy through the inhibition of lysosomal proteases and autophagosome-lysosomal fusion events has attracted further interest in cancer treatment^40^. In our present study, the incubation of bladder cancer cells with CQ increased ICAM-1 expression in all analyzed bladder cancer cells. It is surprising, as our previous study revealed that silencing of ICAM-1 in T24 and HTB9 cell lines inhibited cancer potential. However, it is in line with literature data, which presents that CQ elevated the expression of ICAM-1 in peripheral mononuclear cells^41^. Interestingly, SFN, which is a well-known natural product that possesses anti-cancer and chemopreventive activities, significantly decreased ICAM-1 expression in all analyzed bladder cell lines. Moreover, its combination with CQ was able to maintain the observed effect only in HTB9 and HT-1376. Since ICAM-1 has been postulated to play a role in EMT progression^4,42^, its association with N-cadherin expression was also assessed. Western blot analysis showed that SFN significantly reduced N-cadherin expression. This is consistent with our previous studies^4^, which show that ICAM-1 silencing is correlated with reduced N-cadherin expression and thus leads to reduced bladder cancer potential. Although in the present study CQ contributed to increased ICAM-1 expression, its antitumor activity was demonstrated by our group in melanoma cell lines^12,43^. Similar to our present data, CQ in melanoma cells affected the downregulation of N-cadherin expression^43^. The reduction of surface N-cadherin expression decreased the number of cells that were able to migrate across an endothelial cell monolayer^23^. Here, the level of N-cadherin expression was also correlated with a decrease in cell migration. However, the most valuable effect on bladder cancer migration was detected after treatment with SFN, which may suggest that decreased expression of both N-cadherin and ICAM-1 is necessary to cause the significant effect.

Similar like melanoma cells, CQ significantly contributed to the reduction in cell viability in T24 and HTB-9 bladder cancer cells, but in all herein-studied cancer cells, the effect was enhanced by treatment with the combination. Although treatment of cells with the combination contributes to the deepening of the effect related to either the disruption of cell migration or the reduction of cell viability, it seems that SFN is more responsible for the processes related to EMT^44^ and cell migration, but CQ influences on cell proliferation. It remains in agreement with data showing that CQ is effective in growth inhibition in a variety of cancers, including melanoma^45^, bladder cancer^9^, and oral squamous cell carcinoma^46^. A decrease in cell survival is related to the inhibition of endogenous autophagy by CQ. Although the anti-cancer potential of CQ seems to be well-recognized, its action may be involved with the several limitations. It turns out, CQ has diverse effects on cancer progression. The inhibition of late autophagy can affect cell survival, as well as antigen processing and presentation in the immune system^47^. It is in line with our data, which indicated that CQ may increased the expression of ICAM-1 in all analyzed bladder cancer cells and/or the increased level of AKT phosphorylation at S473 in HT-1376 cell lines. Based on this we postulated that the role of CQ in the regulation of bladder cancer may function dually. Pro-survival autophagy shows function in increasing the progression of bladder cancer and reducing their response to therapy^48^. Aggressive cancer with high levels of autophagy also tends to develop drug resistance by using autophagy to escape drug-induced stresses. However, pro-death autophagy enhances apoptosis and other cell death mechanisms in bladder cancer. Thus, pharmacological modulation of autophagy emerges as a viable strategy to impede bladder cancer progression. Inhibition of autophagy may increase the apoptotic efficiency of drugs. This suggests that the effect (pro- or anticancer action) of compounds which regulate autophagy in cancer e.g. CQ strongly depends on the molecular background of analyzed cancers. In cells with a specific molecular background, the influence of any compounds which regulate autophagy in cancer e.g. CQ may contribute to the stimulation of some signaling pathways associated with cancer progression. However, this not exclude CQ in anticancer therapy, thus the use of chloroquine in combination with other chemotherapeutic reagents may enhance cancer treatment^49^. Similar, SFN may contribute to increased AKT phosphorylation at S473, which has been described in human colon cancer cell lines^50^. On the other hand, sulforaphane treatment lowered the levels of AKT S473 in acute lymphoblastic leukemia^51^ cells and breast cancer cells^52^. Thus, deeply understanding of the particular compounds, mechanisms used in anti-cancer therapy, seems to be important and may prevent possible resistance. Another premise that CQ in T24 and HT-1376 may increase the tumorigenic potential of the analyzed cells is its influence on decreasing activity of GSK-3β kinase. GSK-3β can suppress the Wnt/beta-catenin pathway by phosphorylating β-catenin, which results in the ubiquitin/proteasome-dependent degradation of β-catenin. On the other hand, CQ in HTB-9 cell line reduced the level of AKT on S473 even more significantly than SFN. However, only in the case of SFN was increased GSK-3β activity observed. Overexpression of constitutively active GSK-3β mutants, in some studies, increased chemosensitivity, cell cycle arrest, and reduced tumorigenicity of breast cancers^53,54^. Pharmacological inhibition of GSK-3β induced EMT and invasion in breast cancer^55^. When GSK-3β is inhibited, β-catenin accumulates, translocate to the nucleus, and binds to TCF/Lef transcription factors to activate gene transcription. Our data showed that SFN increased GSK-3β activity, as well it contributed to decrease the level of β-catenin on S552 and increase its phosphorylation level on S33/37; T47. The first effect decreased the β-catenin transcriptional activity^56^, however, the second one leads to β-catenin degradation by the ubiquitin/proteasome pathway. Interestingly, β-catenin silencing is reported to increase the expression of LC3, a major activator of autophagy^57^. It has been reported that glucose deprivation triggers PKC-dependent β-catenin proteasomal degradation, which induces autophagy^58^. The above mechanisms seem to be consistent with the SFN, however SFN alone did not affect autophagy in bladder cancer cells. However, a combination of SFN with CQ increases the effect of CQ on inhibition of autophagy. It seems to be in line with the statement that the role of autophagy in cancer development and therapy appears to be paradoxical and depends on the context^59^. Moreover, the phosphorylation of mTOR S2448 was increased in the case of T24 and HT-1376 after treatment with CQ and SFN alone, and in HTB9 after treatment with SFN in combination with CQ. We focused on S2448 because phosphorylation at this site is implicated in mTORC1 activity and suppression of autophagy^28^ Phosphorylated at S2448 mTOR inhibits the induction of autophagy^60^. The presented above effect not only described the situation after treatment with CQ, but also depicted treatment with SFN. It is surprisingly, because many studies have demonstrated the inhibited effect effects of SFN on Akt/mTOR signaling pathway. Nevertheless, in this context the results are not consistent and even opposite. Some researchers have shown that SFN inhibits tumorigenesis by suppressing this pathway^44,61^, while others demonstrate the activation of SFN on it ^62,63^. After mTORC1 is activated, a series of cascade effects will be activated to inhibit autophagy, what was observed. In this process active mTORC1 may phosphorylate ULK on S757 to prevent ULK interaction with and activation by AMPK. It seems to be in line with our data which revealed the correlation between the activity of mTOR and phosphorylation of ULK S757. However, it should be emphasis that the activity of the signaling pathway is differ in all analyzed bladder cancer cells. The interesting situation was observed in the case of ULK S555, because it is known that phosphorylation of ULK1 at S555 by AMPK, is used as a marker of high autophagy. However, in the case of HTB9 our data demonstrated even the autophagy is inhibited the phosphorylation level of ULK S555 was increased.

The expression of LC3-II correlates with a number of autophagosomes^23^. Here it was revealed that expression of LC3-II undergoes degradation in autolysosomes when treating cells with CQ, which inhibits lysosomal activity, and arrests the latter step of autophagy, as observed by increased level of LC3-II and p62/SQSTM. The role of p62 in multiple solid tumors has been demonstrated, highlighting its oncogene effects in ROS generation and cell growth^64^. On the other hand, p62 silencing in HK-2 cells increases ROS levels, which further indicates the protective role of p62 under oxidative stress^65^. In all analyzed bladder cancer cells, p62 expression level was in greater or lesser extent related with depression of ROS level. Interestingly, SFN in T24 bladder cancer cells significantly increased level of ROS generation, what is in agreement with^66^. Authors demonstrated also that SFN treatment has been associated with loss of the mitochondrial membrane potential in bladder cancer cells. These findings have been interpreted such that SFN triggered ROS generation modifies the intrinsic apoptotic pathway. The other data revealed that SFN induces apoptosis through ROS-dependent disruption of mitochondrial membrane integrity in HTB9 cells^67^. Our results indicate a only a slight increase in ROS level after SFN treatment in the HTB9 line, what indicates that differences in the response of cells to ROS generation may depend on the SFN dose used. Therefore, it seems that ROS exerts its effects on apoptosis via different mechanisms, depending on the cell line. This means that depending on the cancer type, SFN may regulate cancer cells motility in bladder cancer patients over different mechanisms and to a differing extent. Similar, in analyzed bladder cancer cell lines differences in responses after CQ or SFN treatment are visible in the expression of cyclinD3, for which a statistically lower expression level was observed in the T24 cell line after SFN treatment and in the HT1376 line after CQ treatment. SFN demonstrate a promising potential as a chemosensitizer and as synergistic agent with other agents like CQ for the treatment and management of cancers such as bladder. It is consistent with he data based on esophageal squamous cell carcinoma, which shown that autophagy inhibitor chloroquine could potentiate antitumor effects of sulforaphane (SFN) by enhancing activation of SFN on caspase pathway^68^. Moreover, SFN-mediated generation of reactive oxygen species (ROS) induces autophagy via ERK activation, what indicate that SFN-induced autophagy might play a therapeutic function also in brain^19^ .

In summary, our data strongly support the view that the response to anticancer therapy in bladder cancer depends on the molecular background. Although the final effect of CQ or SFN seems to be similar, different, sometimes opposing signaling pathways are activated in the cell. This seems to be important in the context of anticancer therapy. Nevertheless, our data strongly suggest that in the line with high level of ICAM-1 (T24 and HTB9^4^) simultaneous inhibition of autophagy and ICAM-1-dependent signaling pathway revealed an anti-tumor effect, whereas in the line with low ICAM-1 expression level (HT-1376^4^), autophagy inhibition shows a protective effect, associated with the maintenance of the tumorigenic pattern.

Additional studies to refine and clearly elucidate mechanisms of SFN activity will help us identify potential prognostic and predictive biomarkers in bladder cancer. There is no doubt that sulforaphane has some anticancer and chemoprotective properties. As a natural product it is cheaper and safer than other synthetic anticancer agents. Its potential as a therapeutic agent will continue to spur research. Future prospects in this area should focused on large-scale clinical trials conducted therapy in combination not only with CQ, but also with other perspective anti-cancer drugs.

## Supplementary Information

Below is the link to the electronic supplementary material.

Supplementary Material 1

Supplementary Material 2

Supplementary Material 3

## Data Availability

Data will be made available upon request to the main author (email: marta.zarzycka@uj.edu.pl).
