# Supplementary material for: Inhibiting autophagy enhances anti-cancer properties of sulforaphane
Source: Sci Rep. 2026 Jan 15;16:5296. doi: 10.1038/s41598-026-35891-x (PMC12880981; doi:10.1038/s41598-026-35891-x)
Supplement: Supplementary file 1 — Supplementary Material 1 [file 41598_2026_35891_MOESM1_ESM.pdf]

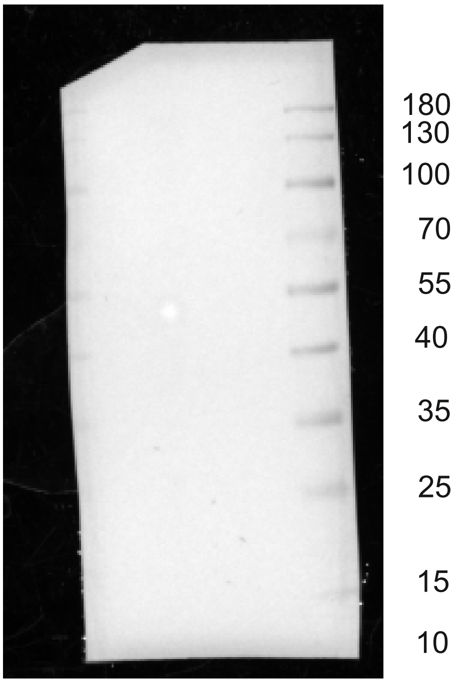

Thermo Scientific™ PageRuler™
Prestained Protein Ladder, 10 to 180 kDa

T24 cell line

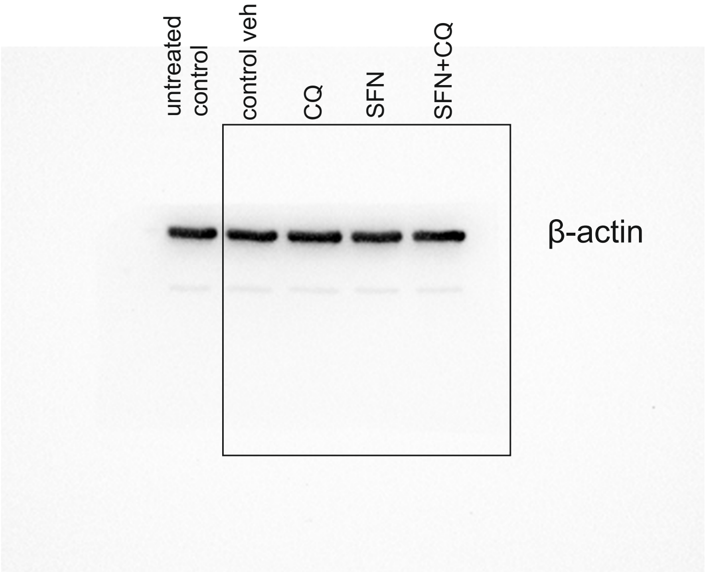

HTB9 cell line

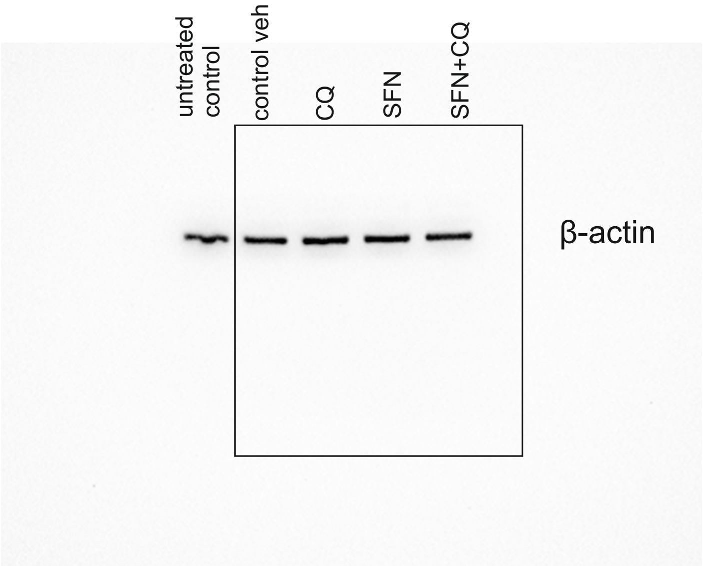

HT1376 cell line

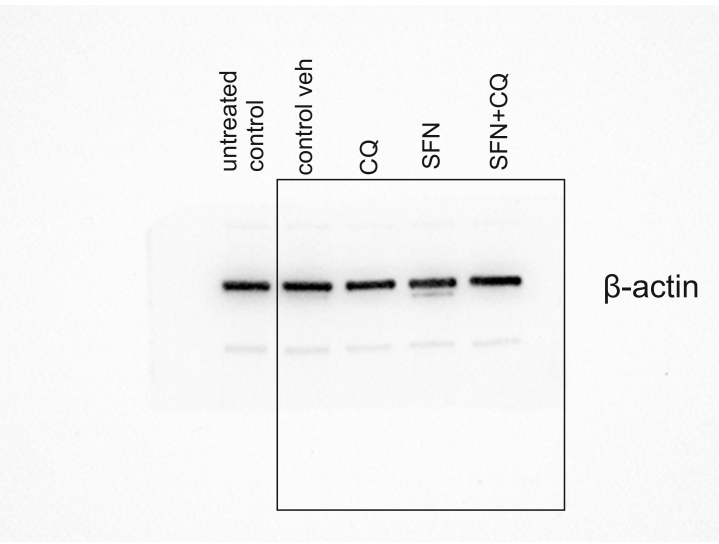

box area indicates the lanes presented in Figures

not applicable — the box area hidden data from an unpresented cell line

T24 cell line

N-cadherin

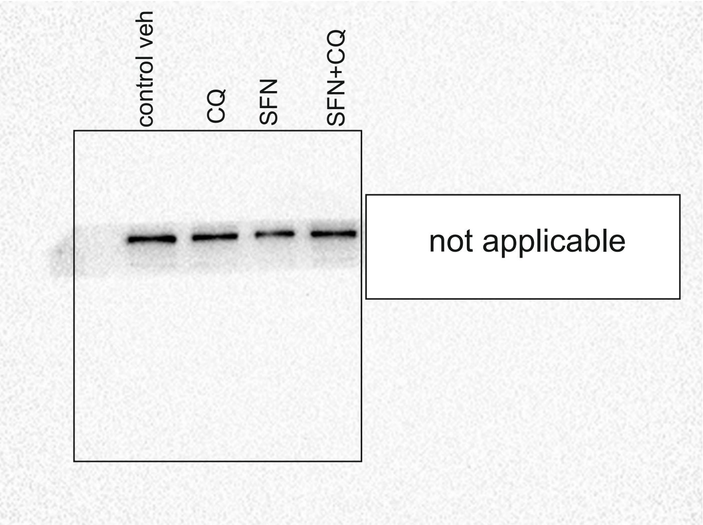

HTB9 cell line

N-cadherin

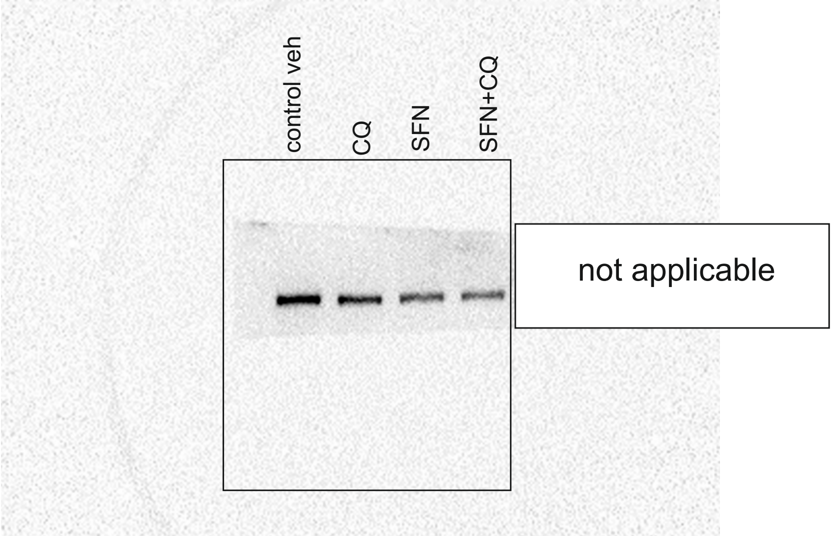

HT-1376 cell line

N-cadherin

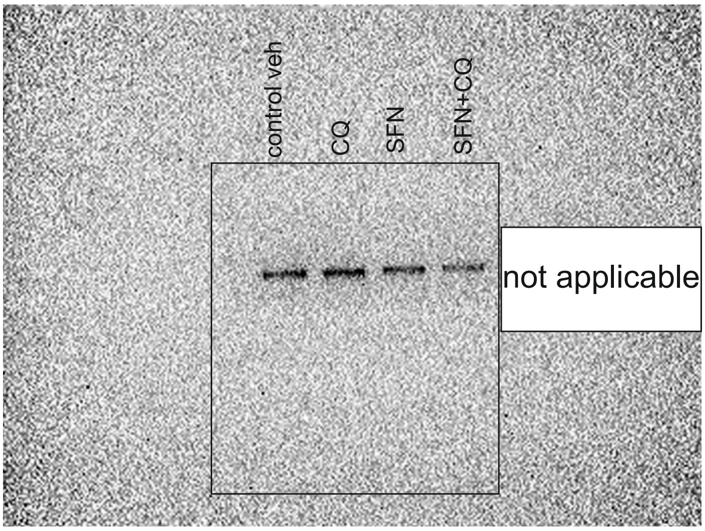

T24 cell line

ICAM-1

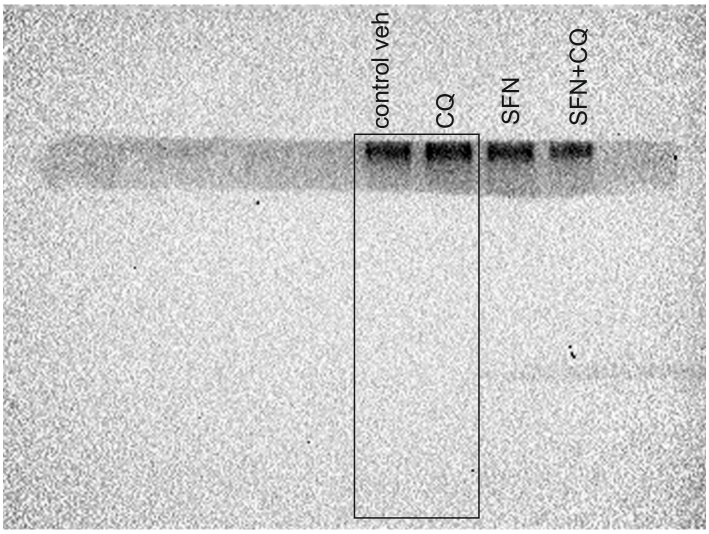

T24 cell line

ICAM-1

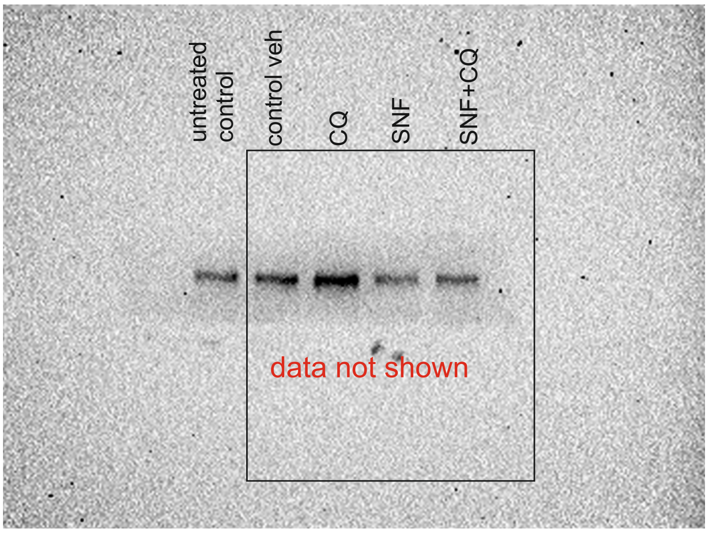

T24 cell line

ICAM-1

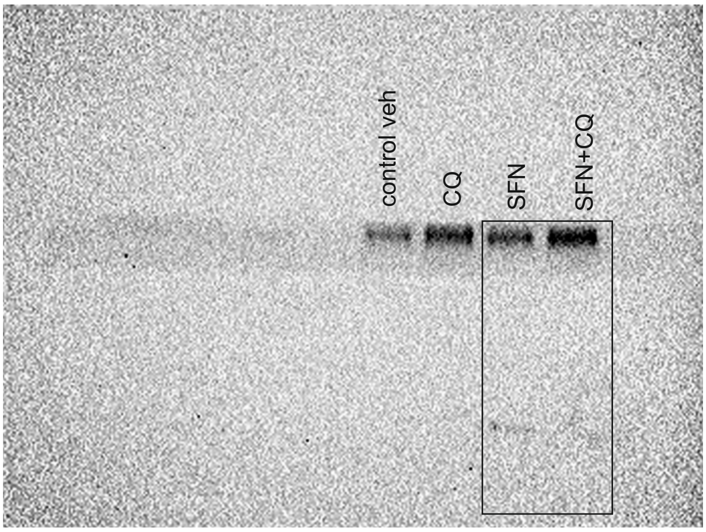

HTB9 cell line

ICAM-1

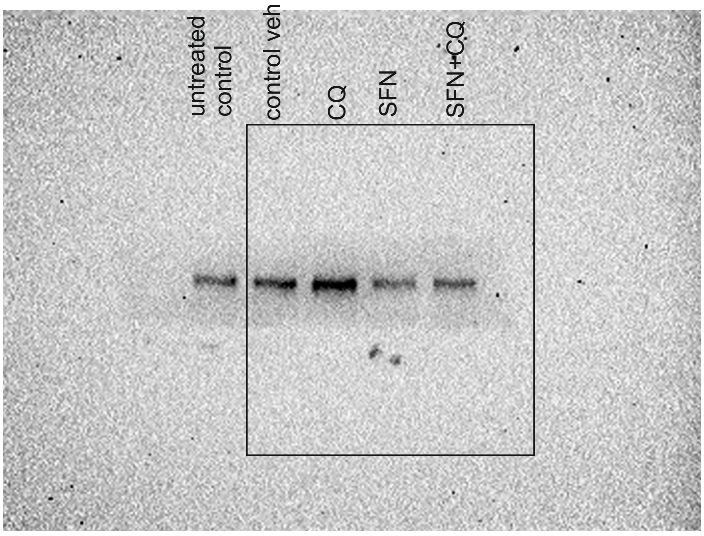

HT-1376 cell line

ICAM-1

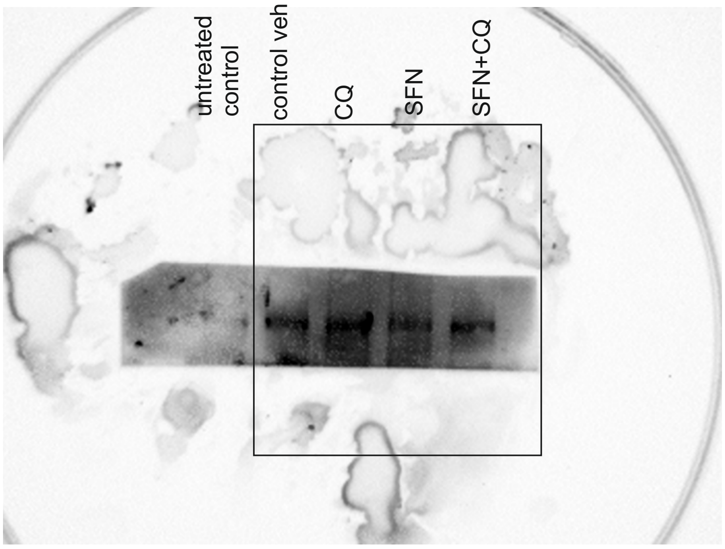

T24 cell line

AKT

HTB9 cell line

AKT

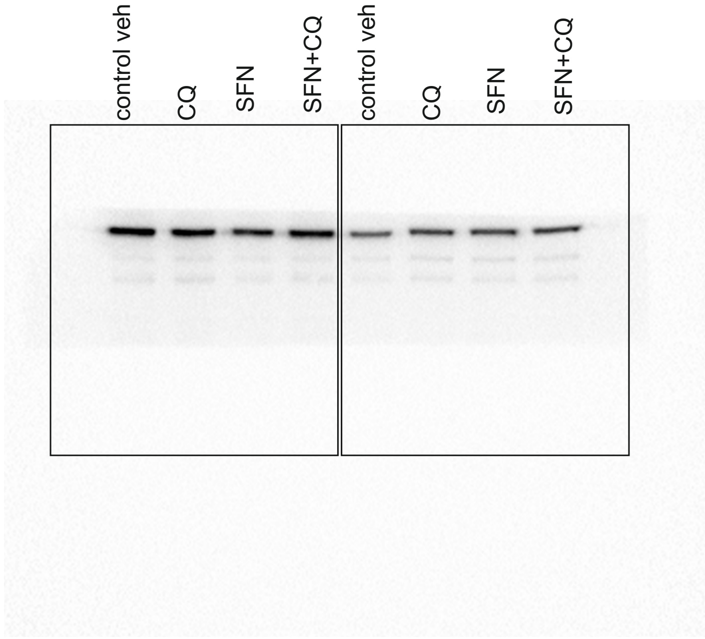

HT-1376 cell line

AKT

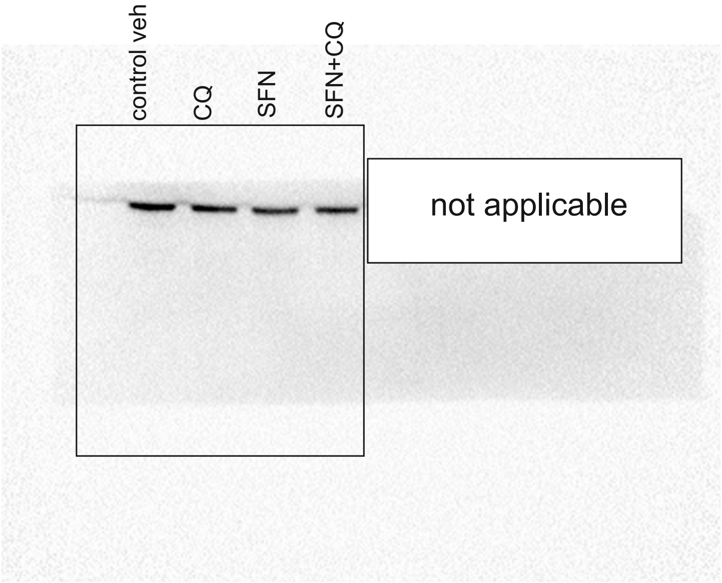

T24 cell line

AKT S473

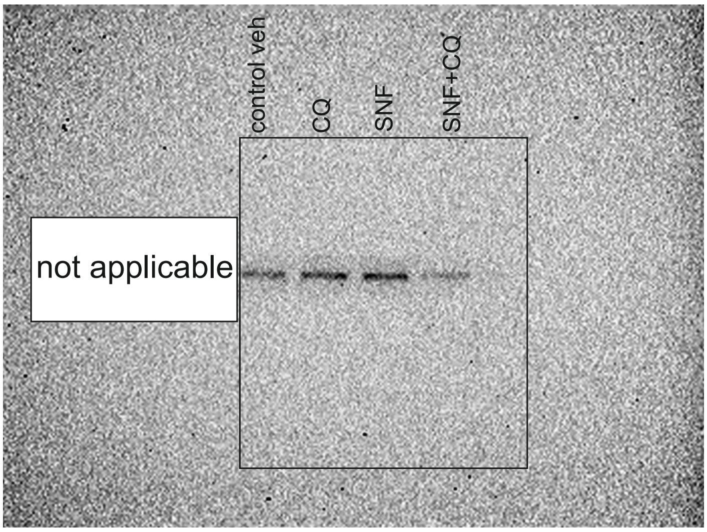

HTB9 cell line

AKT S473

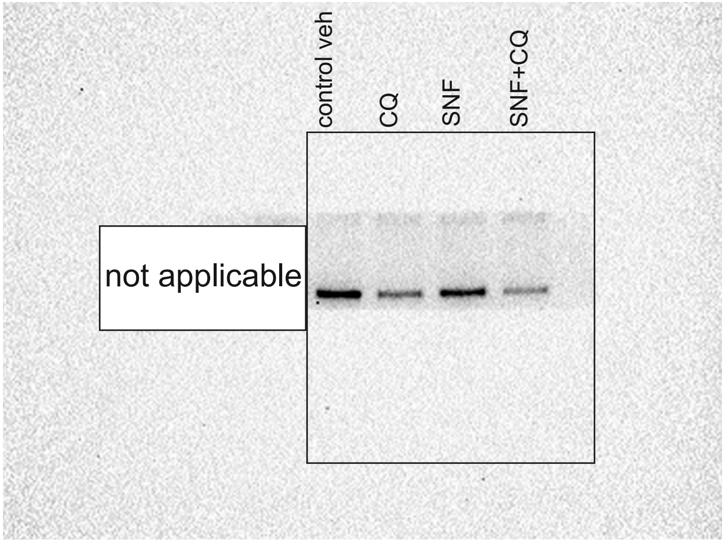

HT-1376 cell line
AKT S473

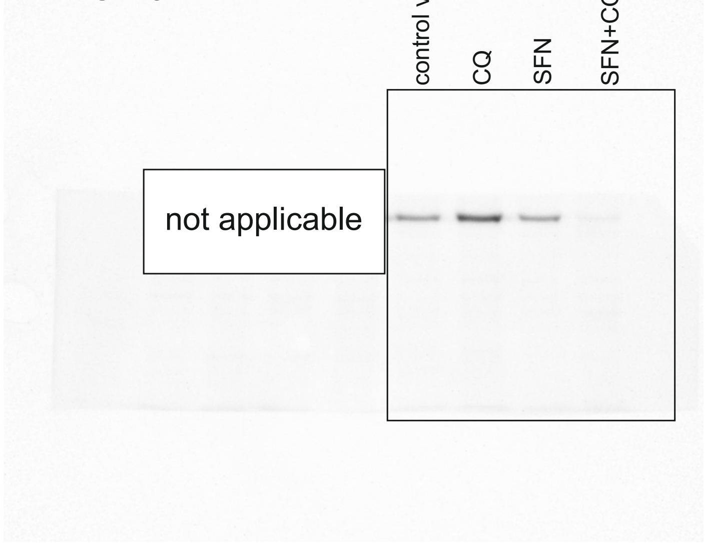

HT-1376 cell line
GSK

HTB9 cell line
GSK

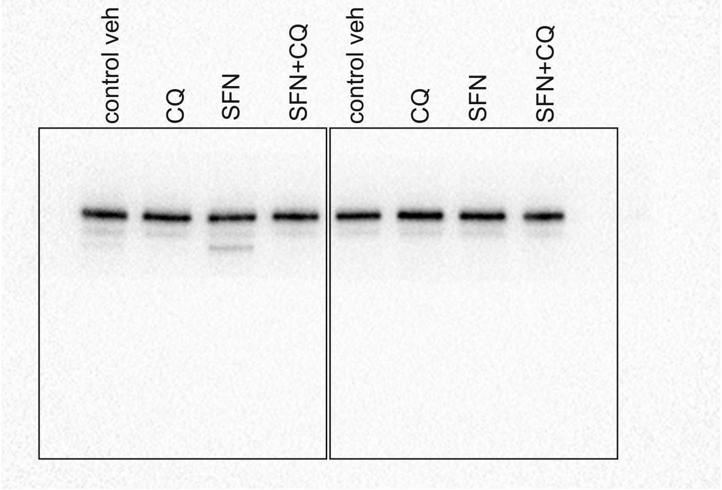

T24 cell line
GSK

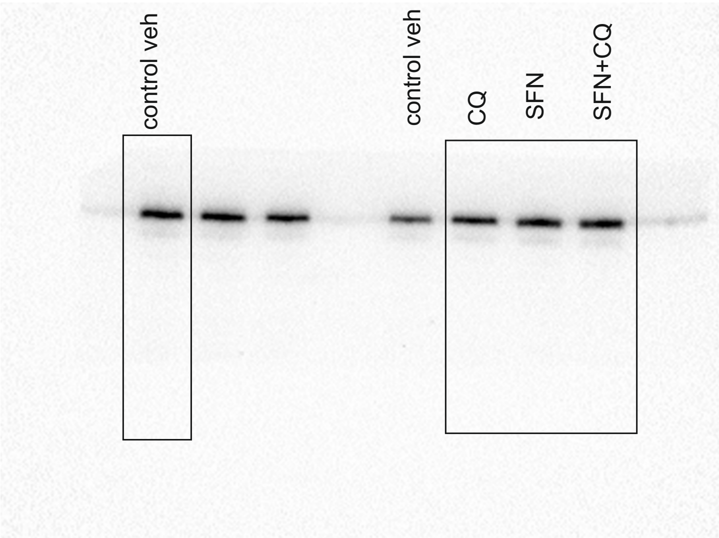

T24 cell line
GSK Y216

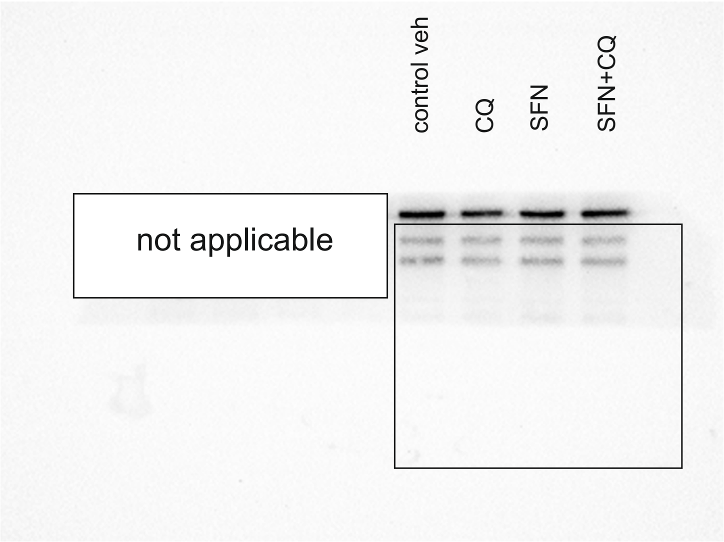

HT-1376 cell line
GSK Y216

HTB9 cell line
GSK Y216

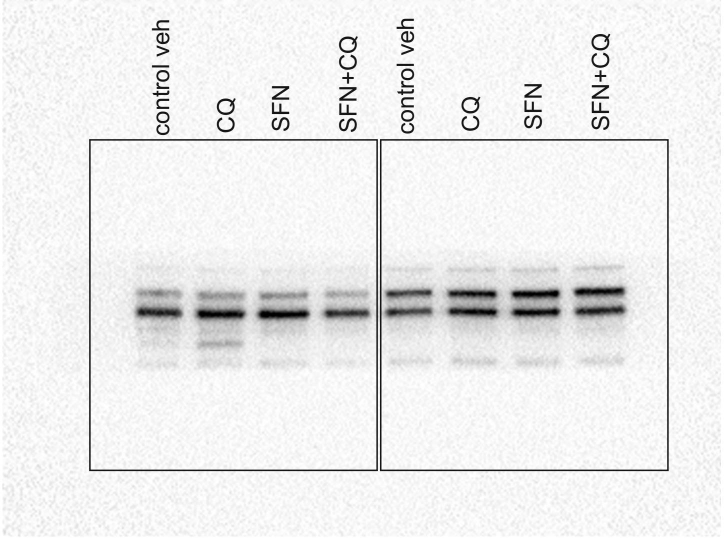

HT-1376 cell line
GSK S9

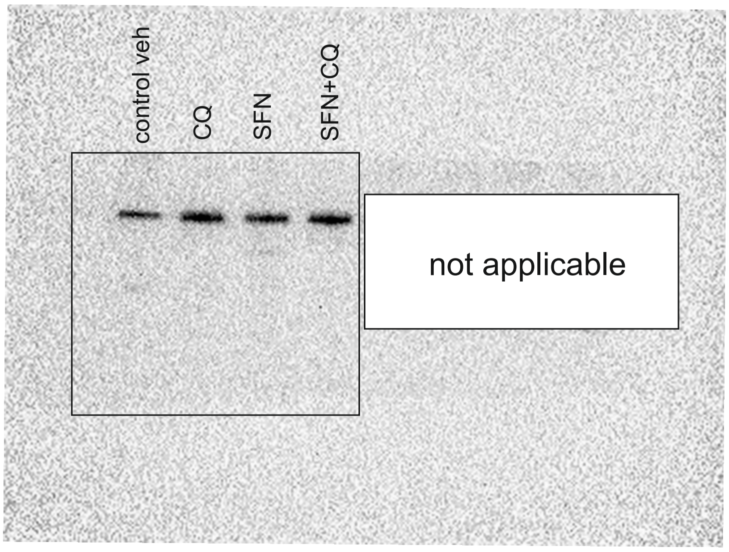

HTB9 cell line
GSK S9

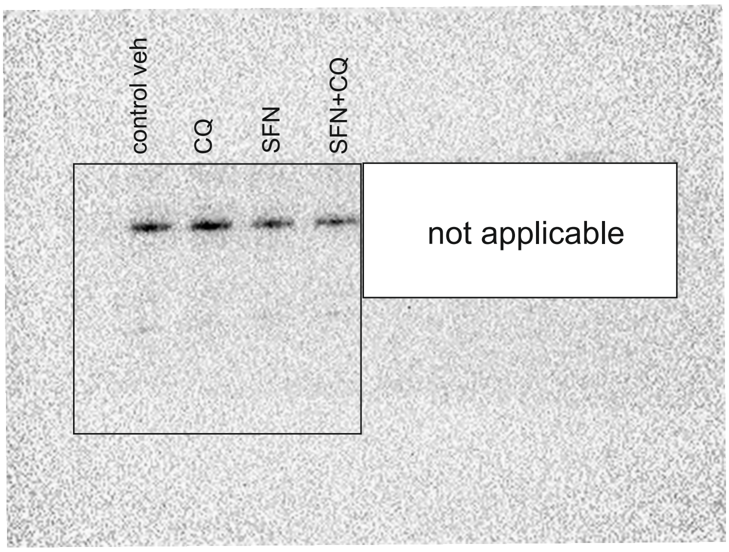

T24 cell line
GSK S9

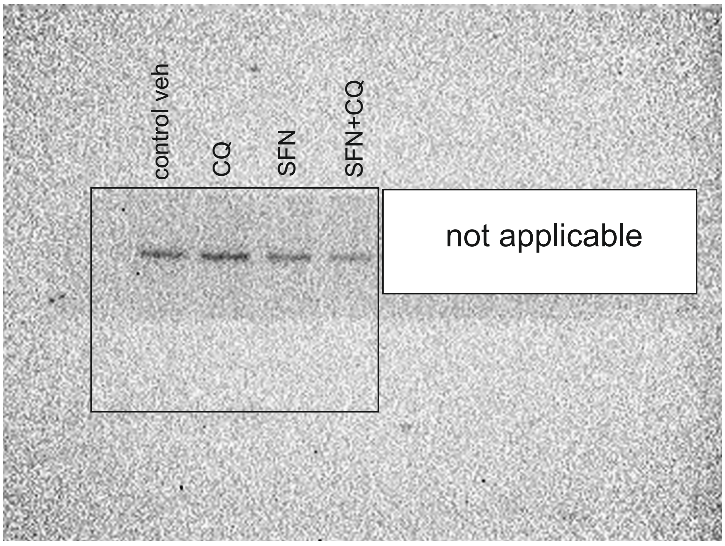

HTB9 cell line
β-catenin

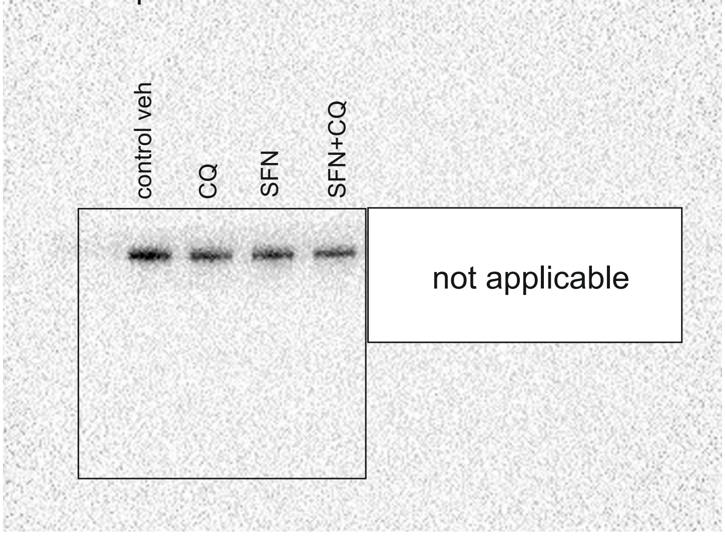

T24 cell line
β-catenin

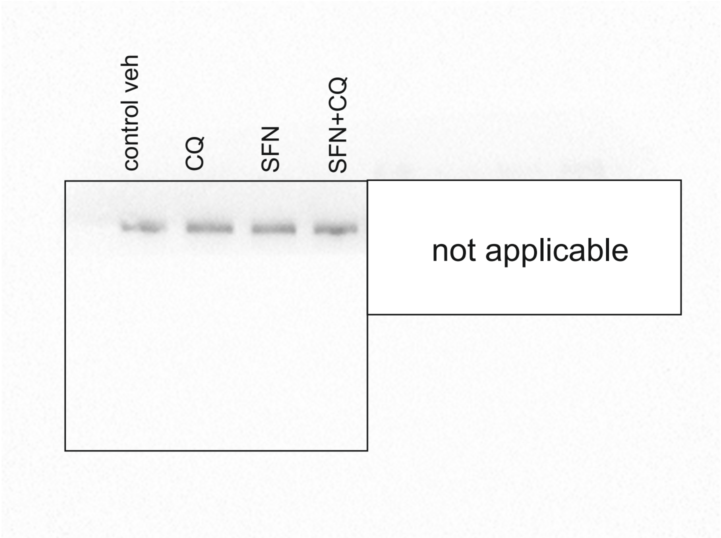

HT-1376 cell line
β-catenin

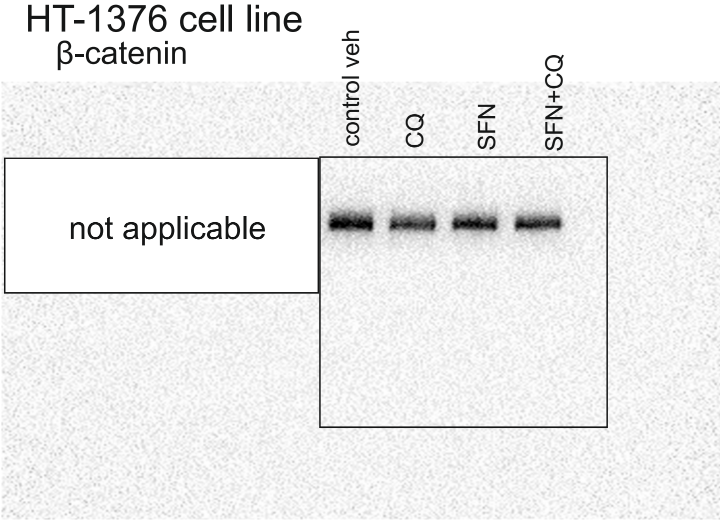

T24 cell line
β-catenin S552

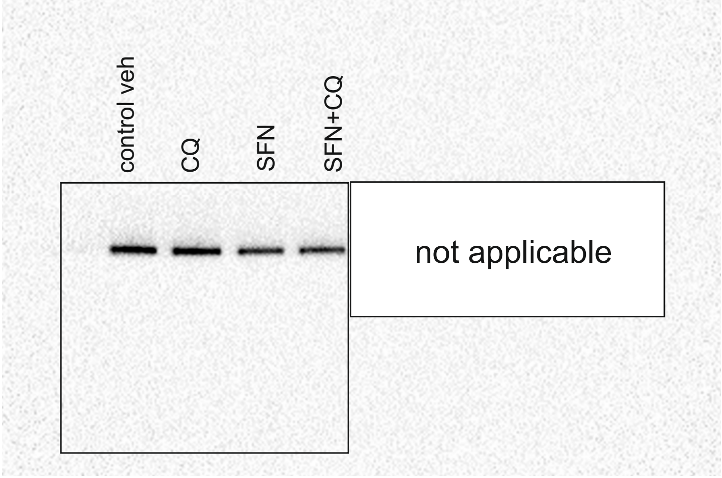

HTB9 cell line
β-catenin S552

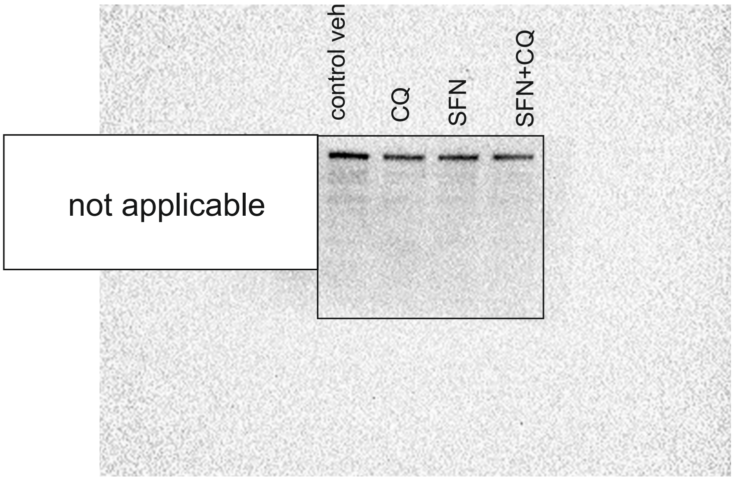

HT-1376 cell line
β-catenin S552

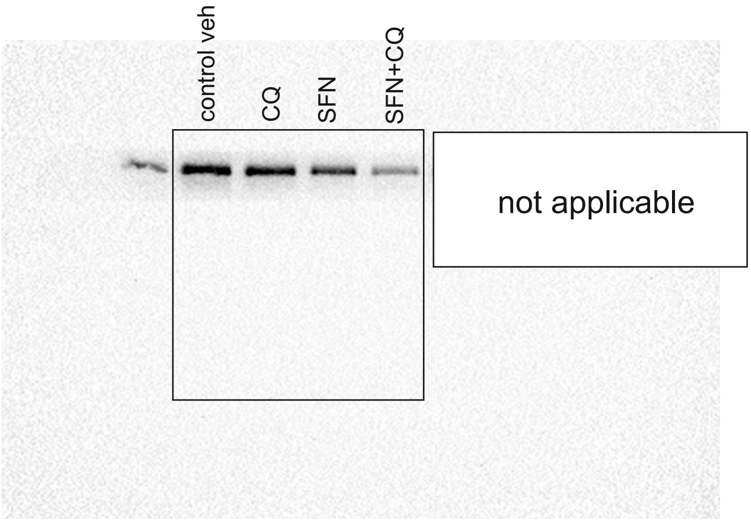

HT-1376 cell line
β-catenin S33/37

T24 cell line
β-catenin S33/37

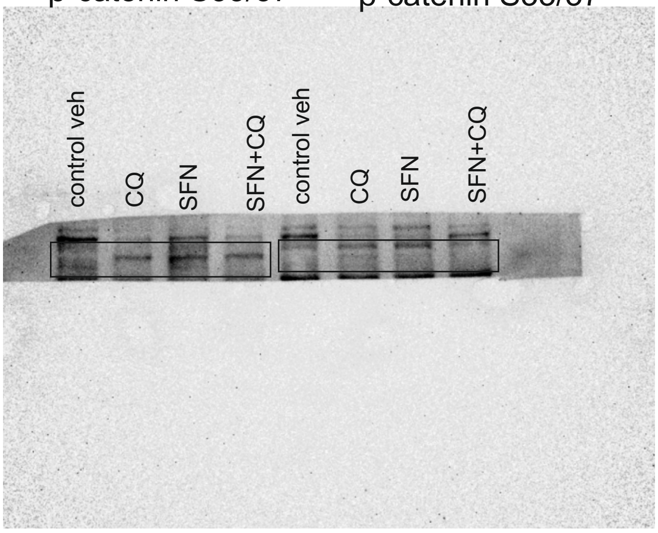

HTB9 cell line
β-catenin S33/37

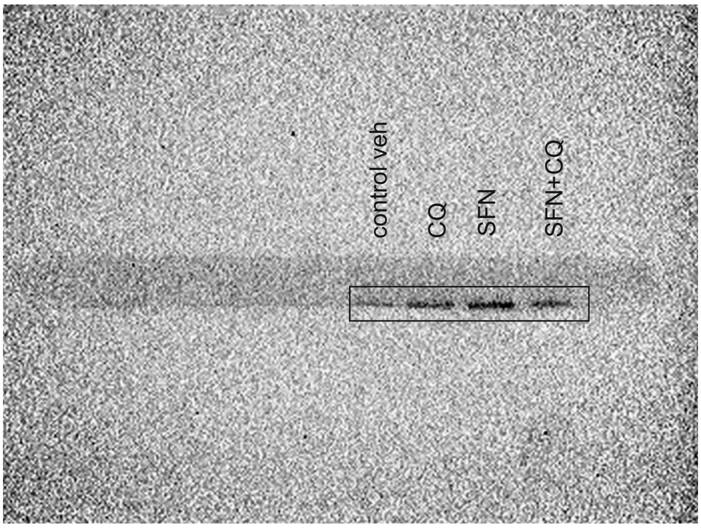

HTB9
mTOR S2448

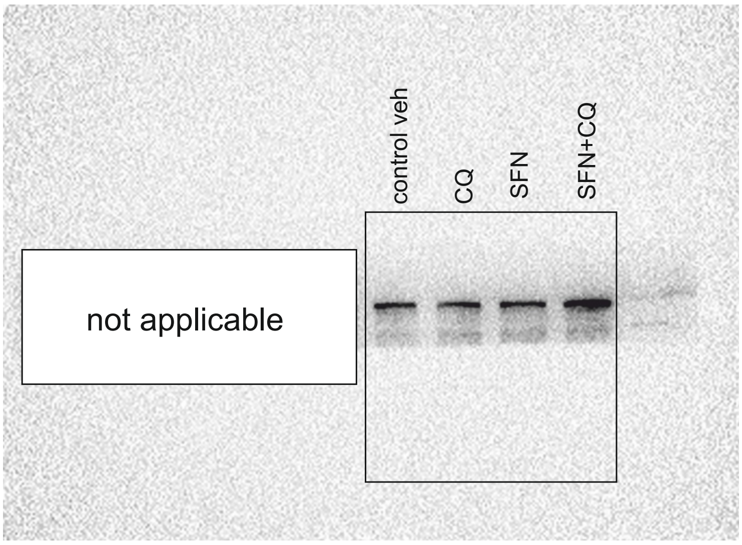

HT-1376 cell line
mTOR S2448

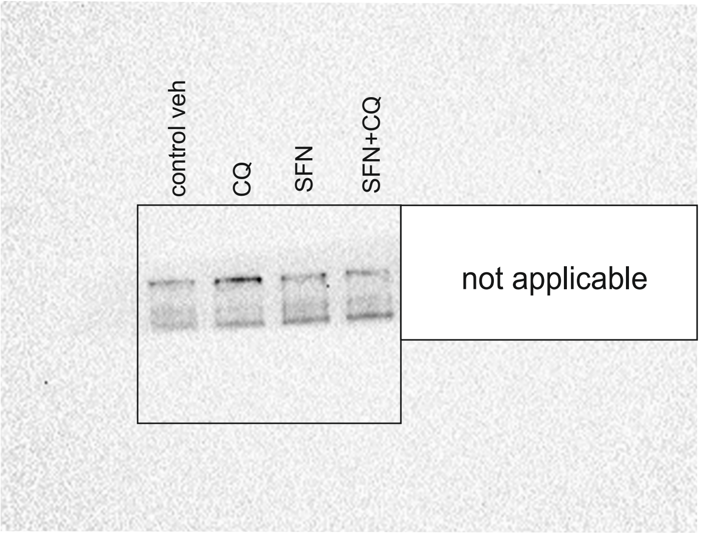

T24 cell line
mTOR S2448

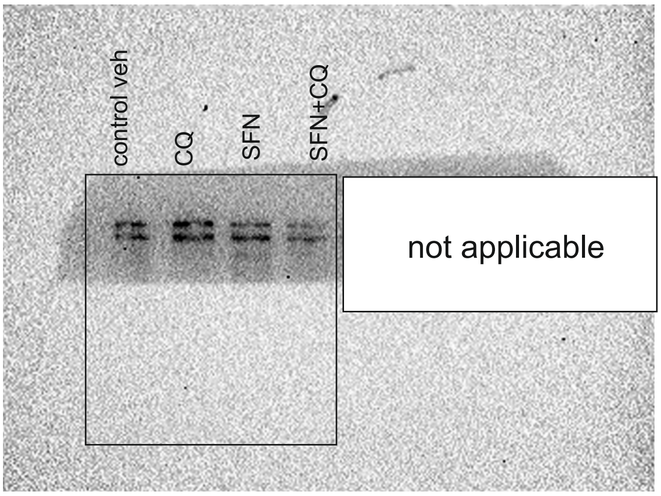

T24 cell line
mTOR

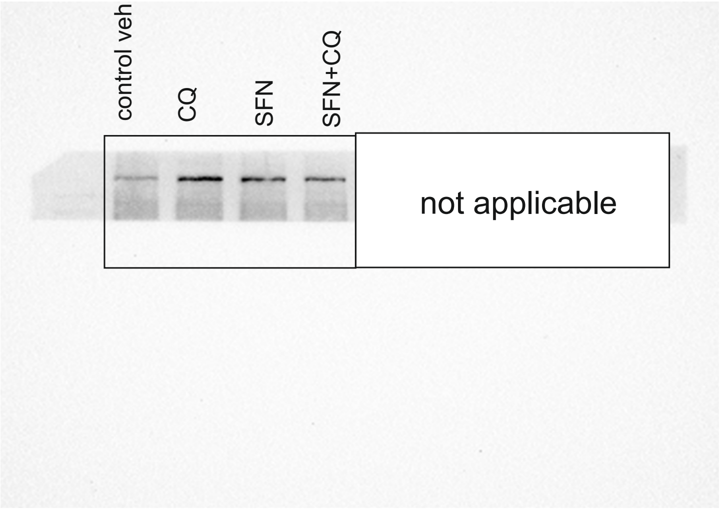

HTB9
mTOR

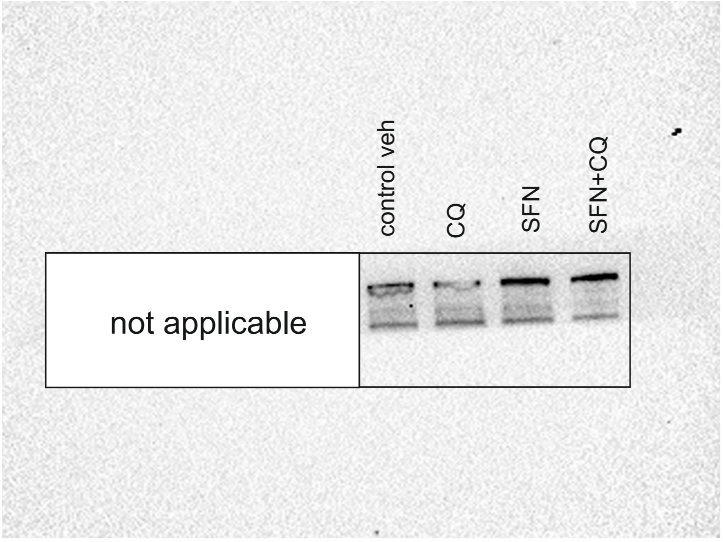

HT-1376 cell line
mTOR

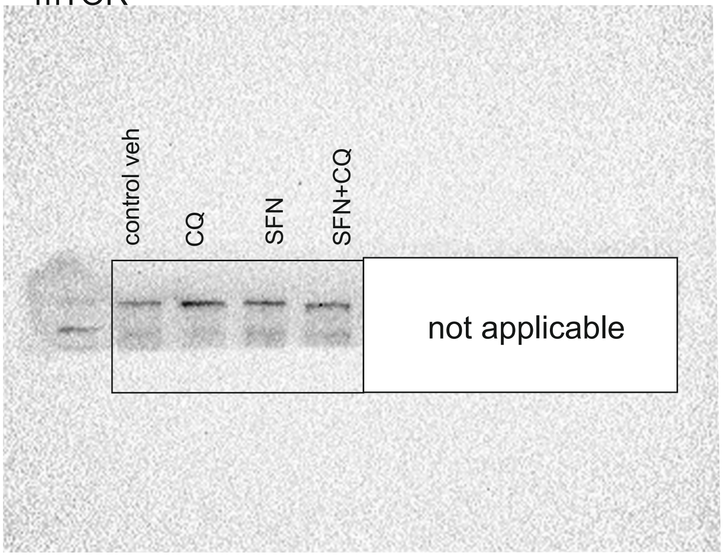

T24
ULK S757

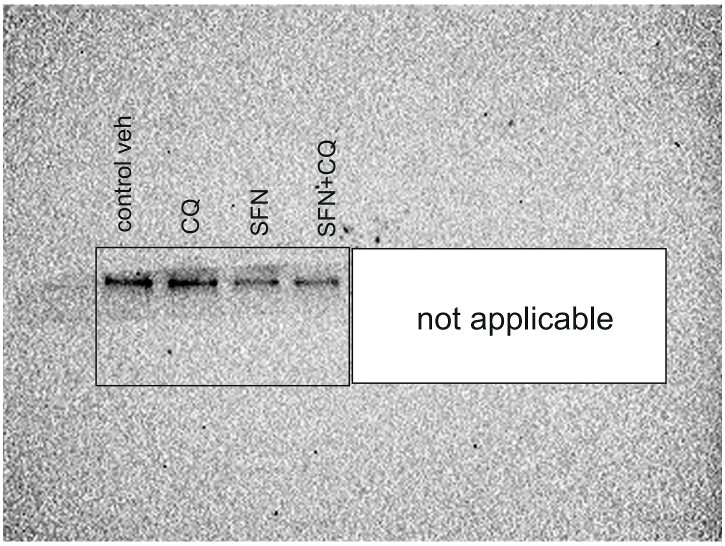

HTB9 cell line
ULK S757

HT-1376 cell line
ULK S757

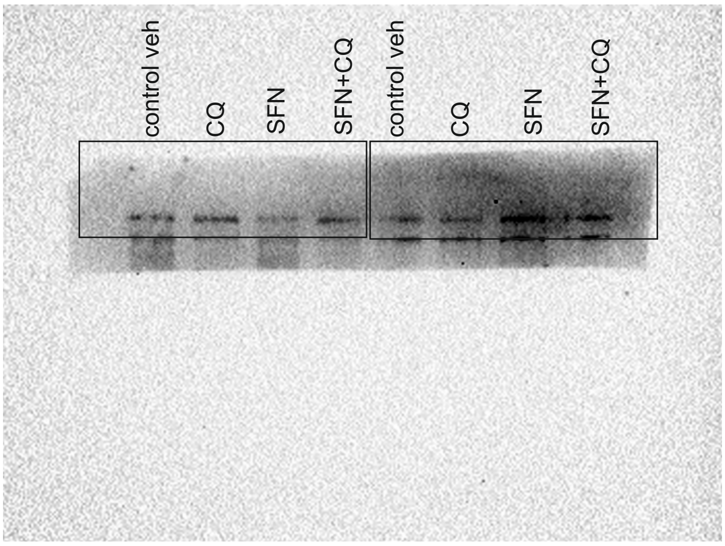

T24
ULK S555

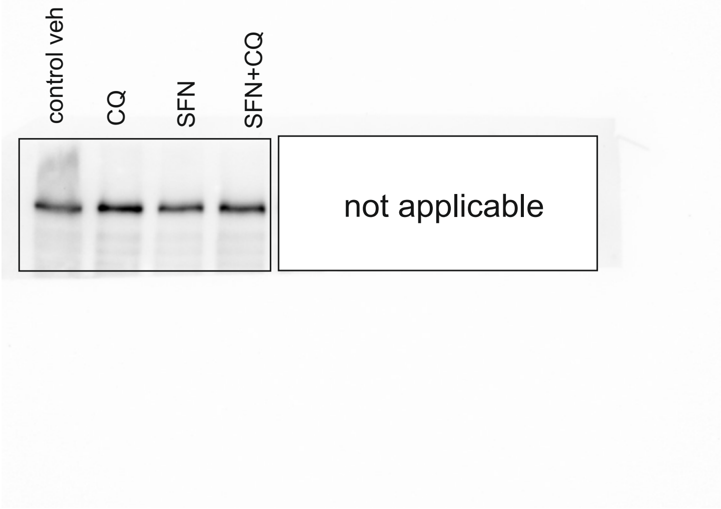

HT-1376
ULK S555

HTB9
ULK S555

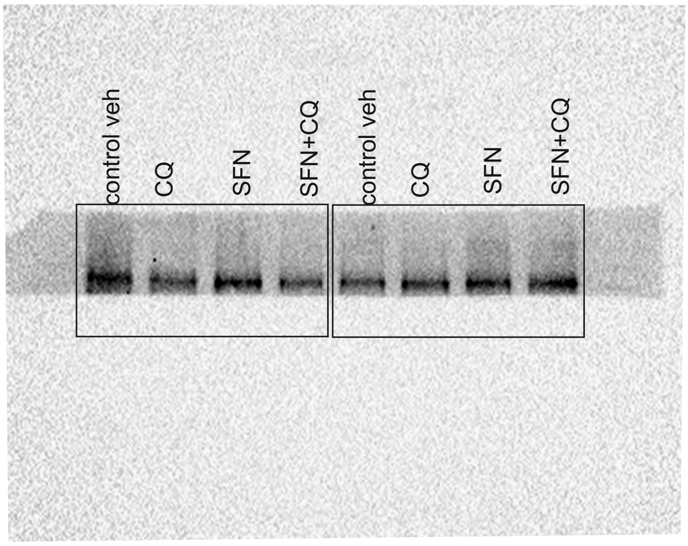

T24
ULK

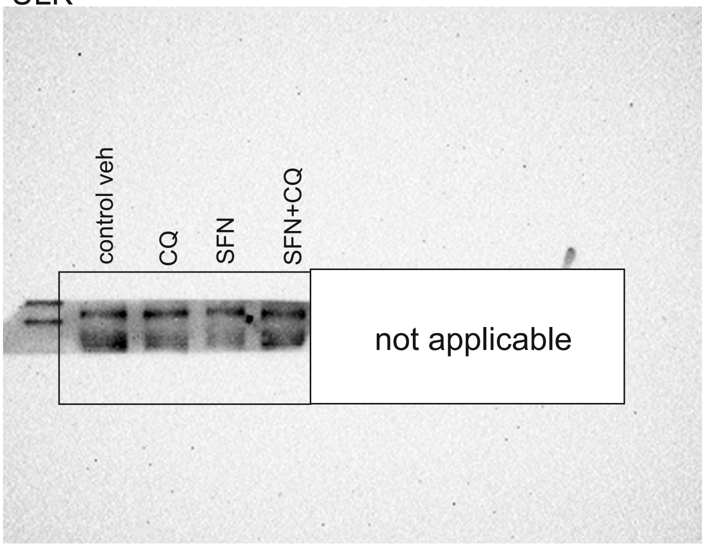

HT-1376 cell line
ULK

HTB9 cell line
ULK

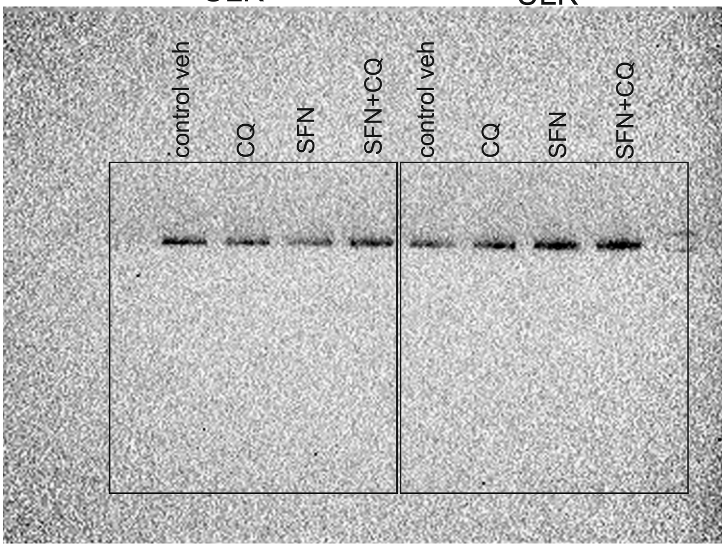

T24 cell line
cyclinD3

HT-1376cell line
cyclinD3

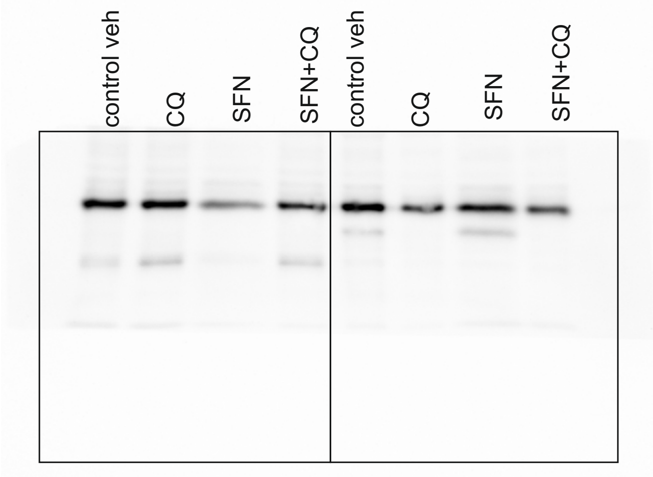

HTB9 cell line
cyclinD3

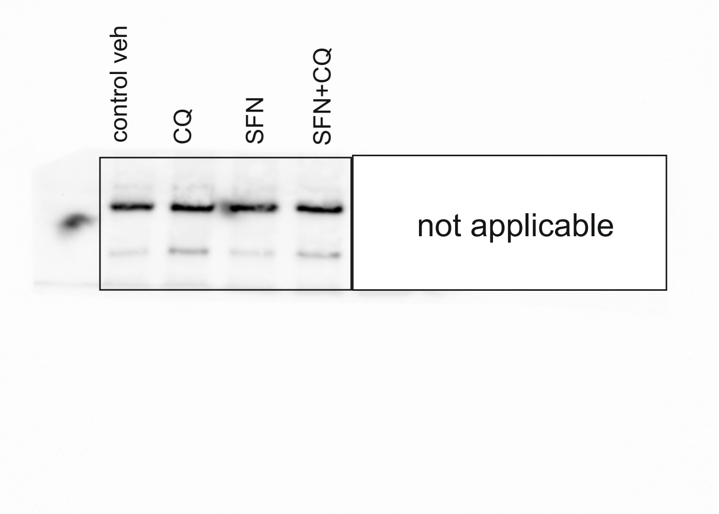

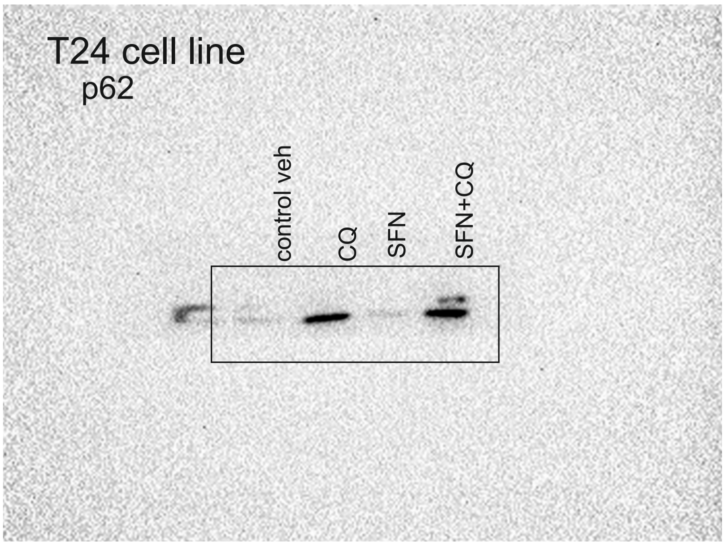
T24 cell line
p62
control veh
CQ
SFN
SFN+CQ

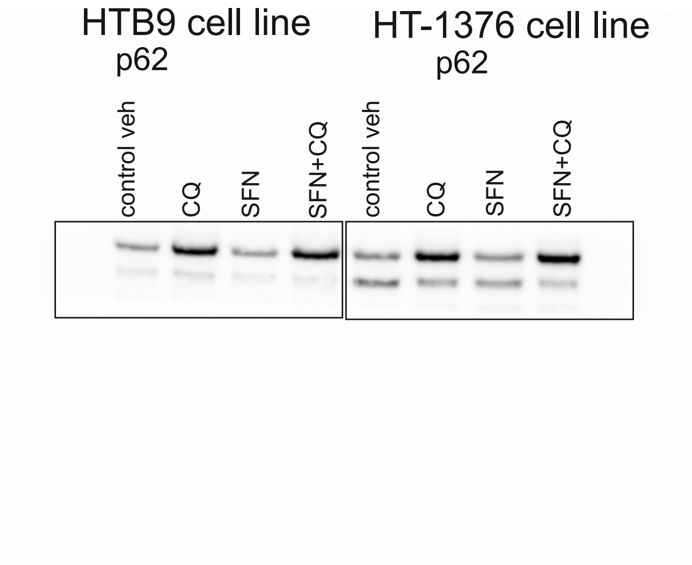
HTB9 cell line
p62
HT-1376 cell line
p62
control veh
CQ
SFN
SFN+CQ
control veh
CQ
SFN
SFN+CQ

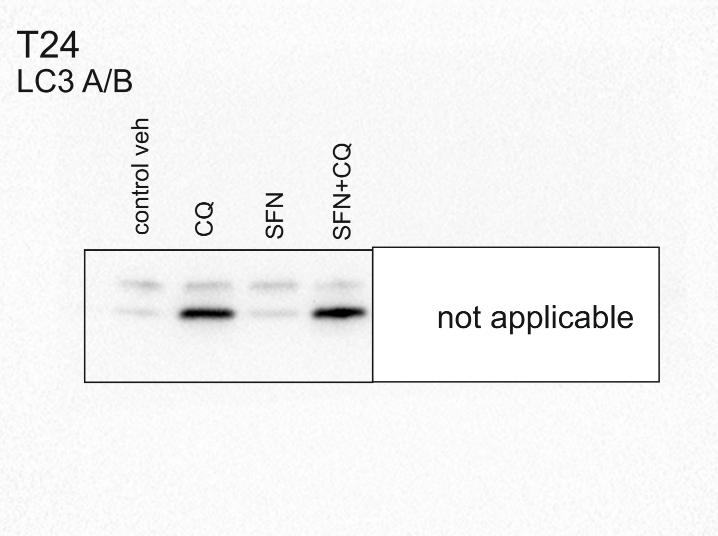
T24
LC3 A/B
control veh
CQ
SFN
SFN+CQ
not applicable

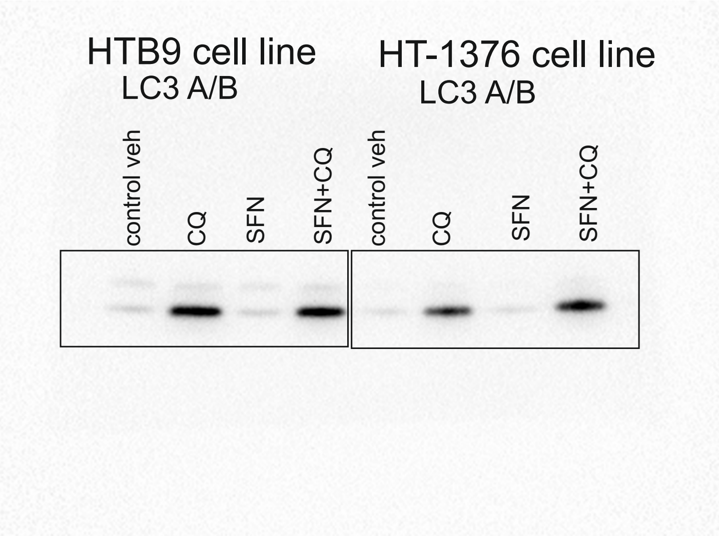
HTB9 cell line
LC3 A/B
HT-1376 cell line
LC3 A/B
control veh
CQ
SFN
SFN+CQ
control veh
CQ
SFN
SFN+CQ
