# Supplementary figures and images for: Inhibiting autophagy enhances anti-cancer properties of sulforaphane
Source: Sci Rep. 2026 Jan 15;16:5296. doi: 10.1038/s41598-026-35891-x (PMC12880981; doi:10.1038/s41598-026-35891-x)

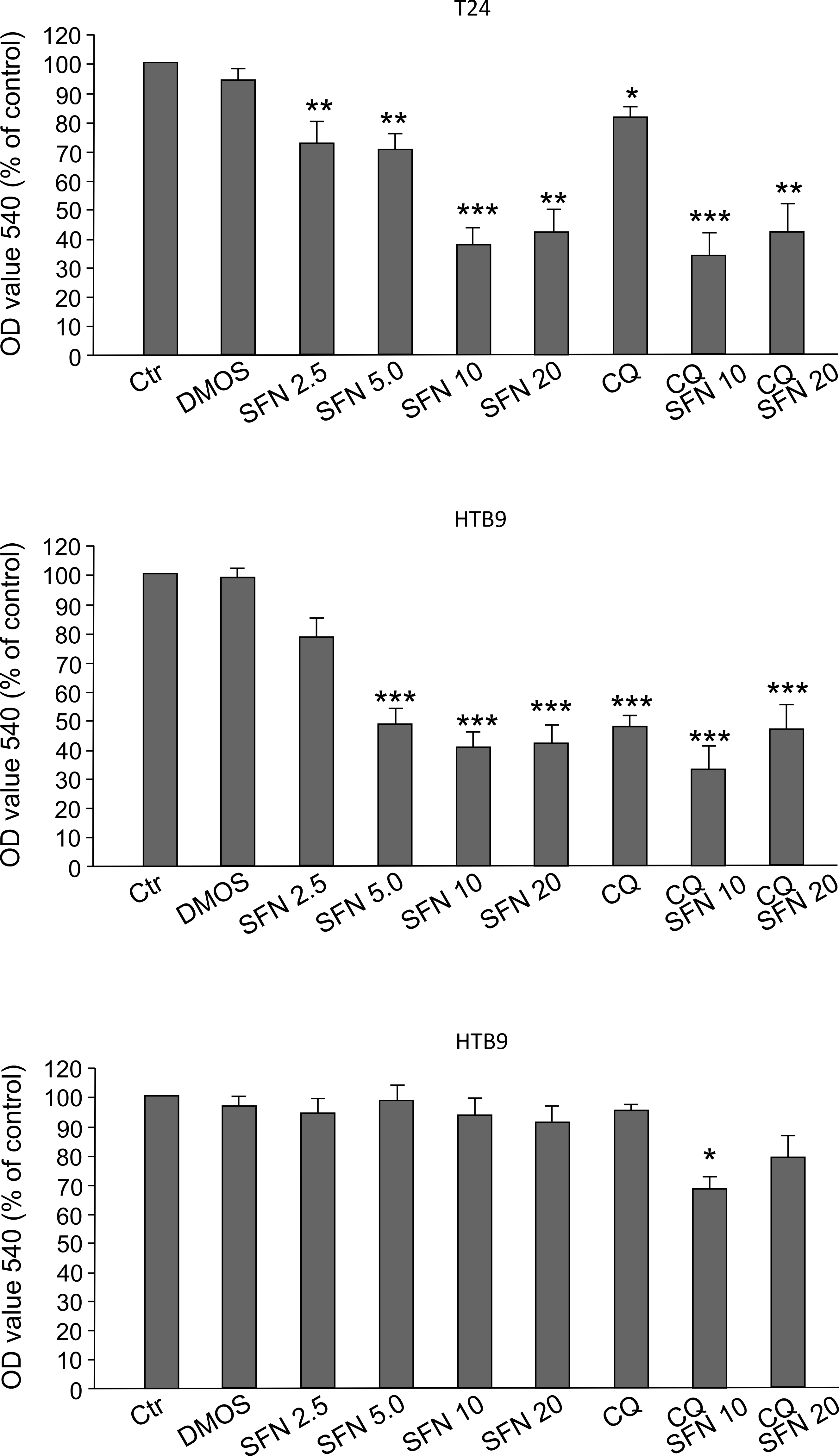

Supplement: Supplementary file 2 — Supplementary Material 2 [file 41598_2026_35891_MOESM2_ESM.tif]
