# Supplementary material for: Inhibiting autophagy enhances anti-cancer properties of sulforaphane
Source: Sci Rep. 2026 Jan 15;16:5296. doi: 10.1038/s41598-026-35891-x (PMC12880981; doi:10.1038/s41598-026-35891-x)
Supplement: Supplementary file 3 — Supplementary Material 3 [file 41598_2026_35891_MOESM3_ESM.docx]

**Supplementary Figure legends**

Supplementary Figure 1. Original blots

Supplementary Figure 2. Effect of Chloroquine (CQ) in the concentration of 50 µM, Sulforaphane (SNF) with different concentrations (2.5 µM, 5.0 µM, 10 µM and 20 µM) or Chloroquine in combination with Sulforaphane (10 µM and 20 µM) on the survival of melanoma cells. Values are expressed as mean ± standard deviation (±SD) in 6 wells in three independent experiments; an asterisk (*) indicates a significant difference: *p < 0.05, **p < 0.01, ***p < 0.001. (A) Cell proliferation was assessed with the crystal violet test. All results are presented as % of control (Ctr).
